# Feasibility of vinegar processing of toxic herbs in Shi–Zao–Tang: toxicity reduction, efficacy preservation in malignant ascites rats and underlying pharmacodynamic mechanisms

**DOI:** 10.1186/s13020-025-01224-9

**Published:** 2025-10-04

**Authors:** Jin-Di Xu, Xiao-Qin Gao, Rong-Ling Zhong, Jing Zhou, Ting Wang, Can Chen, Wei-Feng Yao, Ting Geng, Yi Zhang, Song-Lin Li, Li Zhang

**Affiliations:** 1 Jiangsu Key Laboratory for High Technology Research of TCM Formulae, National and Local Collaborative Engineering Center of Chinese Medicinal Resources Industrialization and Formulae Innovative Medicine and Jiangsu Collaborative Innovation Center of Chinese Medicinal Resources Industrialization, Nanjing University of Chinese Medicine, No. 138, Xianlin Road, Qixia District, Nanjing, 210023 People’s Republic of China; 2 Department of Pharmaceutical Analysis and Metabolomics, Jiangsu Province Academy of Traditional Chinese Medicine, No. 100 Shizi Street Hongshan Road, Nanjing, 210028 People’s Republic of China; 3 Taizhou Key Laboratory for Development of Traditional Chinese Medicine Health Products, Taizhou Engineering Research Center for Quality and Industrialization of Traditional Chinese Medicine, Nanjing University of Chinese Medicine Hanlin College, No. 6, Kuangshi Road, Pharmaceutical High-tech District, Taizhou, 225300 People’s Republic of China

**Keywords:** Shi–Zao–Tang, Malignant ascites effusion, Euphorbiae Pekinensis Radix, Kansui Radix

## Abstract

**Background:**

Shi–Zao–Tang (SZT), a classical formula of Traditional Chinese Medicine orally used for treating malignant ascites effusion (MAE), is made by mixing the powder of Kansui Radix (KR), Euphorbiae Pekinensis Radix (EPR) and fried Genkwa Flos with the decoction of Jujubae Fructus. According to Chinese Pharmacopoeia, vinegar-processed KR and EPR should be used in oral administration. However, toxicity and efficacy of SZT containing vinegar-processed KR and EPR (VSZT) versus SZT in MAE rats, and the potential mechanisms of VSZT against MAE, remain unknown. Here, we comparatively studied the quality, toxicity and efficacy of SZT and VSZT, and explored the potential mechanisms of VSZT against MAE.

**Methods:**

Main components in SZT and VSZT were quantified by liquid chromatographic coupled with triple quadrupole tandem mass spectrometry. The intestinal toxicity and efficacy of SZT and VSZT were comparatively investigated in MAE rats. Specially, intestinal toxicity was evaluated by intestinal barrier function, histopathology and oxidative damage. The efficacy was investigated by amount of ascites, indices in excretion, intestinal motility and inflammation. The potential mechanisms of VSZT treats MAE were explored through integration of metabolomics, 16S rRNA and Western blotting analysis.

**Results:**

VSZT contains less 3-*O*-*EZ* and more ingenol than SZT. VSZT showed reduced intestinal toxicity than SZT in MAE rats. Both SZT and VSZT indiscriminately decreased the amount of ascites and ascitic inflammatory cytokines, promoted urination and defecation, increased fecal water content and intestinal motility. VSZT reversed endogenous metabolism and gut microbiota disorders, down-regulated colonic cAMP, PKA, p-CREB/CREB and AQP3, as well as mesenteric p-VEGFR2/VEGFR2, p-SRC/SRC, and p-VE-cadherin/ VE-cadherin in MAE rats.

**Conclusion:**

VSZT preserved the efficacy of SZT on MAE with lower intestinal toxicity. VSZT increased water excretion and decreased MAE formation to alleviate MAE through regulating gut microbiota, restoring tryptophan and tyrosine metabolism disorders, and affecting cAMP-PKA-CREB-AQP3 and VEGFA-VEGFR2-SRC-VE-cadherine pathway.

**Supplementary Information:**

The online version contains supplementary material available at 10.1186/s13020-025-01224-9.

## Introduction

Malignant ascites effusion (MAE), which is common in advanced malignancy such as ovarian cancer (37%), liver-biliary and pancreatic tumors (21%), and gastric cancer (18%), is a poor prognostic indicator of cancer patients and has a detrimental effect on quality of life and survival of patients [1]. In traditional Chinese medicine (TCM), it is classified under the categories of “Tan Yin” and “tympanites”, both representing syndromes characterized by a disruption in the body fluid homeostasis. It is caused by abnormal accumulation of fluid in the peritoneal cavity and is usually associated with the obstruction of veins and lymphatic vessels by tumor cells, as well as promoted angiogenesis and increased vascular permeability by vascular endothelial growth factor (VEGF) secreted by tumor cells. Besides, inflammation-associated peritoneal hyperpermeability is a direct cause of MAE. Attributed to the increased amount of ascites, the peripheral circulation failed and the renal blood flow and urine output reduced which would aggravate MAE [2]. Patients with MAE usually exhibited constipation caused by gastrointestinal dysmotility which would also further aggravate MAE [3]. Furthermore, when a large amount of ascites produced, gut microbiota homeostasis is disturbed, thereby activating intestinal innate lymphocyte immune system and increasing the release of inflammatory factors. At the same time, intestinal mucin and tight junction proteins are destroyed, the intestinal barrier function is impaired, and intestinal permeability increases, resulting in the translocation of intestinal bacteria, thus further accelerating the progression of the disease [4–6].

Currently, MAE treatments are mainly divided into systemic therapy, which includes chemotherapy and diuretic therapy, and local therapy, which includes puncture drainage and intraperitoneal perfusion therapy. However, due to poor patient compliance, high toxicity and side effects, infection risk, or high treatment costs, and given that these methods can only temporarily alleviate clinical symptoms rather than prevent the generation of MAE [6], additional strategies are needed to effectively treat MAE. It is well known that TCM has obvious advantages in the treatment of difficult and complicated diseases due to its multi-component, multi-target and multi-pathway action. Recently, the use of TCM in MAE treatments has attracted more and more attention [7, 8].

Reportedly, MAE is defined as the peritoneal fluid collection containing cancer cells in a cancer patient and positive ascitic cytology was one of the golden standards for the diagnosis of MAE [6, 9]. Thus, the occurrence and progression of MAE are closely linked to cancer cells invading into the peritoneal cavity. Then, researchers had attempted to intraperitoneally inject Walker 256 cells into rats and found that ascites could be produced 5–7 days later [10]. Therefore, this model had been successfully replicated and applied by some scholars to research on the efficacy of TCM in treating MAE [11]. We also utilized this model to investigate the efficacy of Kansui Radix (KR, radix of *Euphorbia kansui* T.N.Liou ex T.P.Wang.) and KR-containing prescription in the treating MAE [12–15].

Shi–Zao–Tang (SZT), first documented in “*Shang han lun*”, is a classical formula of TCM and specifically applied to expel water and dissipate dampness in diseases characterized by impaired body fluid homeostasis. Now, SZT is usually used for treating malignant pleural effusion (MPE), MAE and cirrhotic ascites [14, 16]. It is made by mixing the powder of KR, Euphorbiae Pekinensis Radix (EPR, radix of *Euphorbia pekinensis* Rupr.) and Genkwa Flos (GF, flower of *Daphne genkwa* Sieb. et Zucc., fried) with the decoction of Jujubae Fructus (JF, fruit of *Ziziphus jujuba* Mill.) [14]. However, its clinical application is seriously restricted due to the toxic herbs in its prescription. For the original prescription of SZT, only GF is explicitly specified to use its processed products. Whether KR and EPR should be processed is not specified. But, according to successive editions of the Chinese Pharmacopoeia and clinical practice, KR should be processed in oral preparations such as pills and powder, and EPR should be processed with vinegar before oral administration [17]. Vinegar-processing could reduce toxicity and preserve efficacy of KR in MAE rats, and the mechanism was related to gut microbiota and its metabolites had been shown before [12, 13]. SZT containing vinegar-processed KR and EPR (VSZT) which replaces raw KR and EPR with their vinegar-processed counterparts, had lower toxicity in normal rats and comparable mitigating effect in MPE rats compared to SZT [16]. Additionally, our results indicated that SZT exerted primarily intestinal toxicity [18]. Besides, SZT has a relieving effect on rats with MAE and FJ enhance the efficacy and attenuate the intestinal toxicity of other herbs in SZT by modulating gut microbiota [14]. However, no investigations were reported on toxicity and efficacy of VSZT compared to SZT in MAE rats and the potential underlying mechanisms of VSZT against MAE.

In the present study, we comparatively studied the main compounds in SZT and VSZT using ultra high performance liquid chromatographic coupled with triple quadrupole tandem mass spectrometry (UPLC-TQ-MS/MS). Subsequently, the intestinal toxicity and efficacy of VSZT and SZT in MAE rats were compared to explore the toxicity reduction with efficacy conservation effect of VSZT. Besides, the potential mechanisms of VSZT against MAE were also preliminary studied. This paper will provide a scientific basis for the safe and effective use of VSZT in clinical practice.

## Materials and methods

### Chemicals and reagents

HPLC-grade methanol and acetonitrile were obtained from Merck (Darmstadt, Germany). MS grade formic acid was purchased from Roe Scientific Inc. (Newark, USA). Ultrapure water was prepared by a Milli-Q system (Millipore, Bedford, MA, USA). Luteolin (**1**), Quercetin (**2**) and Kaempferol (**3**) were obtained from Sichuan Weikeqi Biological Technology Co., Ltd. (Chengdu, China). Genkwanin (**5**) was obtained from Chengdu Pufei De Biotech Co., Ltd. (Chengdu, China). Kansuinin C (**6**), Kansuinin A (**8**), Kansuinin E (**9**), Kansuiphorin C (**10**), Eupha-8,24-diene-3*β*,11*β*-diol-7-one (**11**), Tirucalla-8,24-diene-3*β*,11*β*-diol-7-one (**12**), Ingenol (**4**), Euphol (**14**) and 3-*O*-(2′*E*,4′*Z*-decadienoyl)−20-*O*-acetylingenol (**13**) were self-made and confirmed by HR-MS, and ^1^H and ^13^C-NMR [19]. Their purities were determined to be higher than 95% by HPLC analysis. Magnesium sulfate was purchased from Nanchang Baiyun Pharmaceutical Co., Ltd. (Nanchang, China). Reference compounds of short chain fatty acids (SCFAs) such as acetic acid (Lot B2122212), propionic acid (Lot D1724108), butyric acid (Lot K1919187), isobutyric acid (Lot K1628021), valeric acid (Lot J1909136) and 2-methylvaleric acid (Lot G1706060) were purchased from Shanghai Aladdin Biochemical Technology Co., Ltd. (Shanghai, China). Isovaleric acid (Lot Q7RHG-ED) was obtained from TCI (Shanghai) Chemical Industry Development Co., Ltd. (Shanghai, China).

### Plant material and preparation of processed products

KR (No. NJUCM-20191127) was collected from Baoji, Shaanxi Province, China and identified by Professor Hui Yan (School of pharmacy, Nanjing University of Chinese Medicine, Nanjing, China). EPR (No. NJUCM-20170616) was collected from Bozhou, Anhui Province, China. GF (No. NJUCM-20180323) was collected from Anguo, Hebei Province, China. EPR and GF were identified by Professor Wei Yue (School of pharmacy, Nanjing University of Chinese Medicine, Nanjing, China). JF (No. NJUCM-2020–09-20-B3) was purchased from Xinxiang, Henan Province, China and identified by Professor Sheng Guo (School of pharmacy, Nanjing University of Chinese Medicine, Nanjing, China).

According to the Pharmacopoeia and our previous studies [17, 20, 21], 1000 g of KR was immersed in 300 g vinegar until the vinegar was fully absorbed, and then stir-fried at 260 °C for 9 min to obtain vinegar-processed KR (VKR). 1000 g of EPR was immersed in 300 g vinegar for 2 h, and then diluted with water to 1.5 L, and continue immersed for another 2 h. Boiled the mixture over a gentle fire until the vinegar was fully absorbed. Taken out the product, cool it to 60%–70% dryness, and then dried it under reduced pressure at 40 °C for 8 h to obtain vinegar-processed EPR (VEPR). GF was heated with mild fire until the color changed to obtain fried GF.

### Preparation of SZT and VSZT solutions for animal administration

830 g of denucleated JF was extracted with water twice and concentrated to 500 mL to obtain the water solution of JF. KR, VKR, EPR, VEPR and fried GF were crushed into powder and sifted through a No. 6 standard sieve, respectively. According to the description of dose documented in “*Shang Han Lun*” and our previous studies [16], a certain amount of mixed powder (KR: EPR: fried GF = 1:1:1) was added into JF water solution to obtain the high-dose (56 mg/mL, SZT-H) and low-dose (28 mg/mL, SZT-L) of SZT solutions, respectively. Similarly, a certain amount of mixed powder (VKR: VEPR: fried GF = 1:1:1) was added into JF water solution to obtain the high-dose (56 mg/mL, VSZT-H) and low-dose (28 mg/mL, VSZT-L) of VSZT solutions, respectively.

Positive drug solution: The magnesium sulfate was accurately weighed and dissolved in water to make a concentration of 0.2 g/mL positive drug solution.

### Comparative assessment of the quality of SZT and VSZT using UPLC-TQ-MS analysis

Reference compounds and internal standard solutions: The reference compounds were accurately weighed and dissolved in methanol respectively to get stock solutions (about 1.0 mg/mL). Then, a certain amount of above solutions were mixed and diluted with methanol to produce appropriate concentrations for analysis. Loratadine dissolved in methanol as an internal standard (IS, 0.5 μg/mL).

Sample solutions: Water solution of JF was lyophilized in a vacuum freeze-drying machine at a temperature of −50 °C and a pressure of 100 Pa for 48 h to obtain lyophilized powder of JF. 7.91 g (equivalent to 45 g of JF) lyophilized powder of JF, 0.5 g KR, 0.5 g EPR, and 0.5 g fried GF powders was weighed, added into a 250 mL conical flask and ultrasonically extracted (80 kHz, 25 °C) with 100 mL methanol for 1 h to obtain the extraction of SZT. Similarly, use VKR and VEPR instead of KR and EPR and perform the same steps to obtain the extraction of VSZT. The extraction was centrifuged at 13,000 rpm for 10 min (4 °C). Then, the supernatant was diluted and filtered by a 0.22 µm PTEE syringe filter. Certain IS solution (20 μL) was spiked to each sample (380 μL) for post-acquisition normalization of UPLC-TQ-MS analysis. Then, UPLC-TQ-MS analysis was conducted and the method was validated to quantitatively analyze the major components in SZT/VSZT samples (Supplementary materials).

### Animals, administration and sample collection

Male Sprague-Dawley (SD) rats (80–100 g) were purchased from SiPeiFu (Beijing) Biotechnology Co., Ltd (Lot.110324221101613154, Beijing, China), and housed at a certified animal experimental laboratory, with a 12 h light/dark cycle. Before the experiment, they were acclimatized to the environment for one week. Animals were allowed free access to food and water. Animal welfare and all experimental protocols were approved by the Animal Ethics Committee of Nanjing University of Chinese Medicine (Ethical approval number: 202007A035).

The experimental design is depicted in Fig. 2A. 42 rats were randomly divided into seven groups (n = 6 per group) including the control group (NC), the model group (M), the positive drug group (P), the low dose (M_RL) and high dose (M_RH) of SZT treatment groups, the low dose (M_VL) and high dose (M_VH) of VSZT treatment groups. Except for rats in NC group, the rats in other groups were all intraperitoneally injected with 1 mL of Walker-256 tumor cells suspension (with a cell concentration of 1 × 10^7^ cells/mL; the cells were purchased from Shanghai BioLeaf Biotech Co., Ltd (Shanghai, China)) each rat to induce MAE rat model based on the previously established method [10, 15]. Drug administration was initiated on the second day after modeling for 7 consecutive days. The rats in NC group were administrated with 0.9% NaCl solution by oral gavage. Those MAE rats were then treated with 0.9% NaCl solution (M group), magnesium sulfate at a dose of 0.2 g/mL (P group), SZT at a dosage of 28 mg/mL (SZT-L) (M_RL group), SZT at a dosage of 56 mg/mL (SZT-H) (M_RH group), VSZT at a dosage of 28 mg/mL (VSZT-L) (M_VL group) and VSZT at a dosage of 56 mg/mL (VSZT-H) (M_VH group), respectively.

On the 5th day after administration, the rats were placed in metabolism cages and the urine and feces within 12 h was collected for the amount of feces, the volume of urine and fecal water content analysis. On the 7th day after administration, fresh stool samples from rats were obtained and immediately stored at −80 °C for further gut microbiota and SCFAs analysis. At the night on the 7th day, the rats were placed in metabolism cages, fasted for 12 h without water deprivation before giving the drugs and the urine was collected, centrifuged and stored at −80 °C until metabolomics analysis. On the 8th day, after administration for 1 h, each rat was orally given 2 mL 10% activated carbon suspension (10 g activated carbon dissolved in 100 mL 5‰ CMC-Na water solution). Half an hour later, the ascites was collected after rats were anesthetized by intraperitoneal injection of 1% sodium pentobarbital (40 mg/kg), weighed, and centrifuged to obtain ascitic supernatant. The blood was collected from the abdominal aorta and the serum was obtained by centrifugation. Then, the rat was dislocated and the small intestine range from pylorus to the ileocecal region was removed to measure the intestinal propulsion rate. Jejunum, colon and mesentery tissues were collected, parts of them were fixed in 4% paraformaldehyde for histological assessment and the remaining parts were immediately frozen in dry ice, and then transferred to −80 °C for further analysis.

### Measurement of the fecal water content and intestinal propulsion rate

The feces were dried, and the fecal water content was calculated as follows:

1 $$W\%=\frac{{F}_{w}-{F}_{d}}{{F}_{w}}\times 100\%$$

In Eq. 1, *W%* is defined as fecal water content, *F*_*w*_ is noted for weight of wet feces, *F*_*d*_ is noted for weight of dried feces.

The small intestine was straightened and laid flat on a white paper. The distance from the pyloric section to the farthest point of activated charcoal reached was measured as the carbon powder propulsion distance, and the distance from the pyloric region to the ileocecal section was measured as the full length of the small intestine. The intestinal propulsion rate was calculated as Eq. 2.

2 $$P\%=\frac{{P}_{d}}{{S}_{L}}\times 100\%$$

In Eq. 2, *P%* is defined as intestinal propulsion rate, *P*_*d*_ is noted for carbon powder propulsion distance, *S*_*L*_ is noted for full length of small intestine.

### Hematoxylin & eosin (H&E) and immunohistochemical (IHC) staining

The jejunum, colon and mesentery were embedded in paraffin and sliced into 5 µm thick section. Sections of jejunum and mesentery were stained with H&E for histopathological examination. Sections of jejunum, colon and mesentery were deparaffinized, antigen retrieved, blocked, incubated with primary antibody (ZO-1 (1:200), occludin (1:200), AQP3 (1:500), VEGFA (1:200) and VEGFR2 (1:200)), incubated with an HRP-labeled secondary antibody, washed and stained with diaminobenzidine (DAB) for IHC analysis. The mean density of the target signals was analyzed by Image Pro Plus 6.0 software (Media Cybernetics, USA).

### Biochemical assay and Enzyme-linked immunosorbent assay (ELISA)

The jejunum was accurately weighed and homogenized in cold saline (w/v = 1:9). Then, the homogenate was centrifuged (3500 rpm, 10 min) to obtain the supernatant for the biochemical assay. The total protein was measured using the BCA kit. Corresponding assay kits were used to measure the levels of SOD, LDH and GSH according to the manufacturer’s instructions, respectively.

The levels of VEGF, TNF-α, IL-6 and IFN-γ in the supernatant of ascites, LPS, DAO, MTL, VIP and SS in serum were quantified using ELISA kits following the manufacturer’s instructions.

### Metabolomics analysis

Serum was precipitated with ice-cold methanol (1:12, v:v), vortexed for 20 s and subsequently centrifuged at 13,000 rpm for 10 min at 4 °C to obtain the supernatant. Similarly, the supernatant of urine was obtained. To ensure data quality, quality control samples (QCs) were prepared by mixing equal amounts of each serum or urine samples, respectively. Then, UPLC-QTOF-MS/MS-based metabolomics analysis method was conducted and validated (Supplementary materials).

The acquired data were analyzed using Progenesis QI v2.0 software (Waters corporation, Milford, MA, USA). After the chromatographic peaks were automatically aligned, extracted and normalized, the resultant data matrices were further imported into EZinfo 3.0 software (Waters corporation, Milford, MA, USA) for Partial least squares-discriminant analysis (PLS-DA) and orthogonal partial least squares discriminant analysis (OPLS-DA). For demonstrating the differential metabolites which contributed to the separation of NC and M group in the score plot generated from OPLS-DA analysis, the metabolites with variable importance in projection value (VIP) > 1 were selected and further verified by Student’s *t*-test (*t*-test) whether there was a significant difference between groups. The FDR control method was employed to effectively manage the false positive rate, with an FDR threshold set at *q* < 0.05 for significance. Then, the metabolites with VIP > 1, *P* < 0.05, *q* < 0.05, and fold change value (FC) ≥ 1.2 or FC ≤ 0.78 were considered as potential biomarkers of MAE. The biomarkers were identified by comparing the data obtained from UHPLC-QTOF-MS analysis with online databases including Human Metabolome Database (HMDB), ChemSpider and Kyoto Encyclopedia of Genes and Genomes (KEGG) databases. Metabolic pathway analysis was performed by using online analytical tools MetaboAnalyst (v5.0) database (https://www.metaboanalyst.ca), SMPDB (https://smpdb.ca/) and PathBank (http://www.pathbank.org/).

### Fecal 16S rRNA sequencing and data visualization

The sequencing of fecal samples was supported by Shanghai OE Biotech Co., Ltd (Shanghai, China). Briefly, total genomic DNA was extracted using MagPure Soil DNA LQ Kit (Magan) following the manufacturer’s instructions. DNA concentration and integrity was measured with NanoDrop 2000 (Thermo Fisher Scientific, USA) and agarose gel electrophoresis. The extracted DNA was used as template for PCR amplification of bacterial 16S rRNA genes. For bacterial diversity analysis, V3-V4 (or V4-V5) variable regions of 16S rRNA genes was amplified with universal primers 343 F (5′-TACGGRAGGCAGCAG-3′) and 798R (5′-AGGGTATCTAATCCT-3′). The final amplicon was quantified using Qubit dsDNA Assay Kit (Thermo Fisher Scientific,USA). Sequencing was performed on an Illumina NovaSeq 6000 with 250 bp paired-end reads (Illumina Inc., San Diego, CA; OE Biotech Company; Shanghai, China). Sequences were performed further denoising as follows: reads with ambiguous, homologous sequences or below 200 bp were abandoned. Reads with 75% of bases above Q20 were retained using QIIME software (version 1.8.0). Then, reads with chimera were detected and removed using VSEARCH software. Clean reads were subjected to primer sequences removal and clustering to generate operational taxonomic units (OTUs) using VSEARCH with 97% similarity cutoff. The representative read of each OTU was selected using QIIME package. The co-occurrence network and spearman correlation coefficient analysis at genus level (|r|> 0.8, *P* < 0.05) were done in R software using the “psych” and “igraph” package. The network diagram was visualized by Gephi (v0.10.1) and the node topological features were calculated. Based on the connectivity values within and between modules, the within-module connectivity and among-module connectivity (ZiPi) analysis were conducted and visualized by ggClusterNet package. The node attributes of the topological structure were classified into four types: module hubs (Zi > 2.5 and Pi < 0.62), connectors (Zi < 2.5 and Pi > 0.62), network hubs (Zi > 2.5 and Pi > 0.62), and peripherals (Zi < 2.5 and Pi < 0.62). Module hubs, connectors, and network hubs were classified as key nodes.

### Determination of SCFAs

According to our previous study [22], the contents of fecal SCFAs were determined (detailed information please see Supplementary materials).

### Western blotting

The mesentery and colon were homogenized in ice-cold RIPA lysis buffer with 1% protease inhibitor cocktail and 1% PMSF, and lysed on ice for 30 min, respectively. Loading buffer was added, and samples were boiled at 100 °C for 5 min to denature protein. Proteins were separated by SDS-PAGE gel, transferred to polyvinylidene difluoride (PVDF) members, blocked for 2 h at room temperature, washed, incubated with anti-VEGFR2 (1:1000), anti-Phosphor-VEGFR2 (Tyr951) (1:500), anti-VE-cadherin (1:1000), anti-VE cadherin (phosphor Y685) (1:500), anti-SRC (1:800), anti-Phosphor-Src (Tyr416) (1:1000), anti-*β*-actin (1:3000), anti-CREB (1:1000), anti-CREB (phosphor S133) (1:5000), anti-PKA (1:1000), anti-cAMP protein kinase catalytic subunit (1:2000), anti-AQP3 (1:1000) and anti-β-tubulin (1:1000) at 4 °C overnight, washed, incubated with secondary antibody for 2 h at room temperature, and washed. Finally, an enhanced chemiluminescence (ECL) solution was applied to the protein bands and the images were obtained using the Tanon 5200 Chemiluminescent Imaging System (Tanon, Shanghai, China).

### Statistical analysis

All the data were expressed in mean ± standard error analyzed by Graphpad Prism. The significance between two groups was evaluated using Student’s *t*-test. The significance of multiple groups was assessed using one-way ANOVA with Dunnett’s multiple comparisons test. *P* < 0.05 was considered as statistically significant. Spearman’s correlation analysis between the differential metabolites and effect-related indices, as well as between differential metabolites and gut microbiota were performed using oebiotech tools at 95% confidence level with *P* < 0.05 and *q* < 0.05 (https://cloud.oebiotech.com).

## Results

### VSZT contained lower levels of more toxic components and higher levels of less toxic ones than SZT

**Fig. 1:**
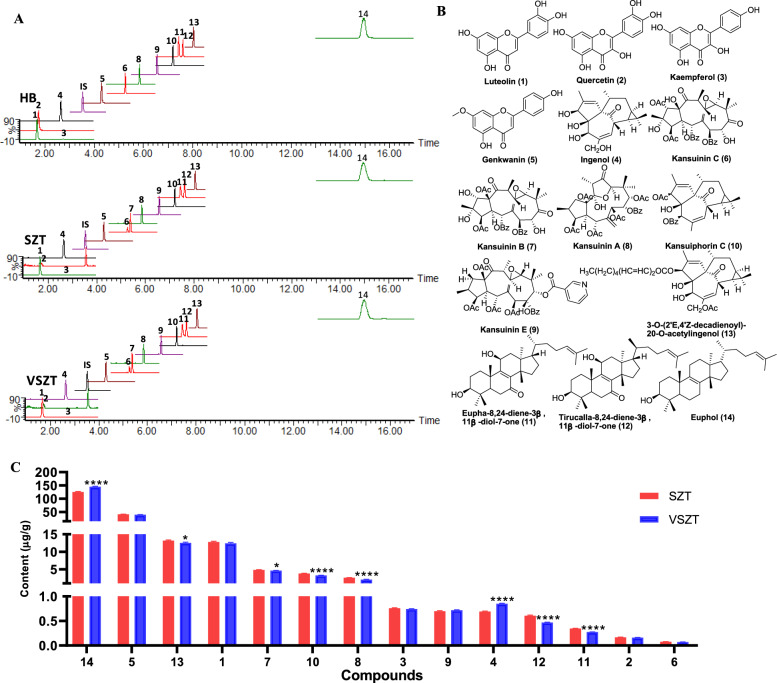
MRM chromatograms, structures and contents of 14 targeted compounds in SZT and VSZT (n = 6)

The contents of 14 main components including 3 ingenane-type diterpenes (compound **4**, **10** and **13** originated from KR), 4 jatrophane-type diterpenes (compound **6**, **7**, **8** and **9** originated from KR), 3 triterpenoids (compound **11**, **12** and **14** originated from KR and EPR) and 4 flavonoids (compound **1**,** 2**,** 3** and **5** originated from fried GF; compound **2** originated from JF) were comparatively quantified in SZT and VSZT. The structures of reference compounds and typical chromatograms of SZT and VSZT were depicted in Fig. 1. The developed method was validated in terms of linearity, sensitivity, precision, repeatability, stability, accuracy and matrix effect (Table S2 and S3). All the analytes showed good linearity in the test range (*R*^2^ ≥ 0.9910). The LODs and LOQs of all the analytes were in the range of 0.4360–7.795 and 0.9020–16.79 ng/mL, respectively. The overall relative standard deviations (RSDs) of precision and repeatability were no more than 9.0% and 14%, respectively. The stability presented as RSDs was within 15% and the accuracy expressed as spiked recoveries ranged from 88.9% to 110.6% with RSDs less than 9.0%. As to matrix effect, evaluated by ion inhibition rates, the results showed that all the analytes were in the range of −14% to 10%. Therefore, the developed method was reliable for quantifying multi-compounds in SZT and VSZT.

Compared with SZT, the contents of compound **7**, **8**, **10**, **11**, **12** and **13** significantly decreased (*P* < 0.05), while compound **4** and **14** significantly increased (*P* < 0.0001) in VSZT (Fig. 1C), indicating that VSZT contained lower levels of more toxic components and higher levels of less toxic ones than SZT.

### VSZT showed lower intestinal injury than SZT in MAE rats

**Fig. 2:**
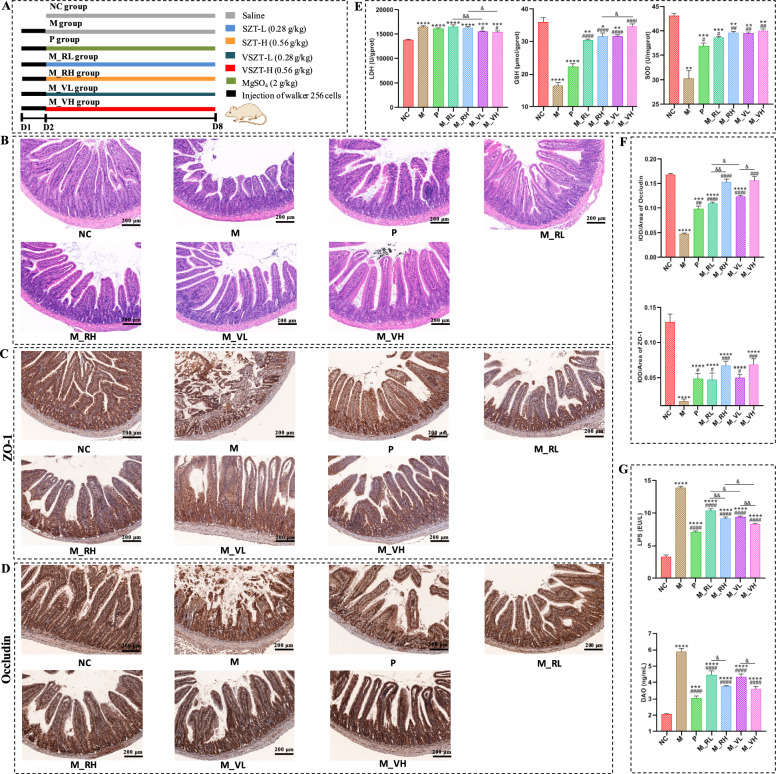
VSZT showed lower intestinal injury than SZT in MAE rats (n = 6). **A** Schematic mode of experimental grouping and administration in rats. **B** Representative histopathological images of the jejunum. **C** Representative immunohistochemical images of ZO-1 in the jejunum. **D** Representative immunohistochemical images of occludin in the jejunum. **E** Levels of GSH, LDH and SOD in the jejunum. **F** Statistical analysis of ZO-1 and occludin in the jejunum. **G** Serum levels of LPS and DAO. NC, control group; M, model group; P, MAE rats administrated with magnesium sulfate at the dosage of 2 g kg^−1^; M_RL, MAE rats administrated with SZT at the dosage of 28 mg mL^−1^; M_RH, MAE rats administrated with SZT at the dosage of 56 mg mL^−1^; M_VL, MAE rats administrated with VSZT at the dosage of 28 mg mL^−1^; M_VH, MAE rats administrated with VSZT at the dosage of 56 mg mL^−1^. Compared with NC group, *P < 0.05; **P < 0.01; ***P < 0.001; ****P < 0.0001; Compared with M group, ^#^*P* < 0.05; ^##^*P* < 0.01; ^###^*P* < 0.001; ^####^*P* < 0.0001; Compared with M_RL or M_RH group, ^&^*P* < 0.05; ^&&^*P* < 0.01

As depicted in Fig. 2B, the jejunal mucosal structure of rats in NC group was intact, with intact and neatly arranged villi and epithelial cells, and no inflammatory cells infiltration. Compared with NC group, rats in other groups exhibited varying degrees of impaired mucosal integrity, exfoliated intestinal epithelial cells and shortened villi. However, compared with rats administered with different doses of SZT, administration of VSZT showed a reduction in intestinal injuries. Oxidative damage of the jejunum was further evaluated (Fig. 2E). Compared with NC group, notably elevated levels of LDH (*P* < 0.001) and significant reduced activity of SOD (*P* < 0.01) and levels of GSH (except for M_VH group, *P* < 0.05) were observed in all the MAE rats. However, compared with groups administered with SZT, the levels of GSH (especially in high-dose group, *P* < 0.05) and SOD were increased with decreased levels of LDH (*P* < 0.05) in groups administered with VSZT. Furthermore, ZO-1 and occludin in jejunum were detected (Fig. 2C, D, F). The results revealed a significant reduced expression levels of occludin (except for M_RH and M_VH group) and ZO-1 in the jejunum of all the MAE rats compared with rats in NC group (*P* < 0.001). Measurements of serum concentration of LPS and activity of DAO indicated that both of the two indicators were significantly increased in all the MAE rats compared with rats in NC group (*P* < 0.001) (Fig. 2G). Interestingly, increased levels of ZO-1 and occludin (especially in low-dose group, *P* < 0.05), and decreased levels of LPS (*P* < 0.05) and DAO were found in the groups given VSZT than SZT. These results showed that VSZT showed lower intestinal injury than SZT in MAE rats.

### VSZT exhibited comparable water-expelling efficacy to SZT in MAE rats

**Fig. 3:**
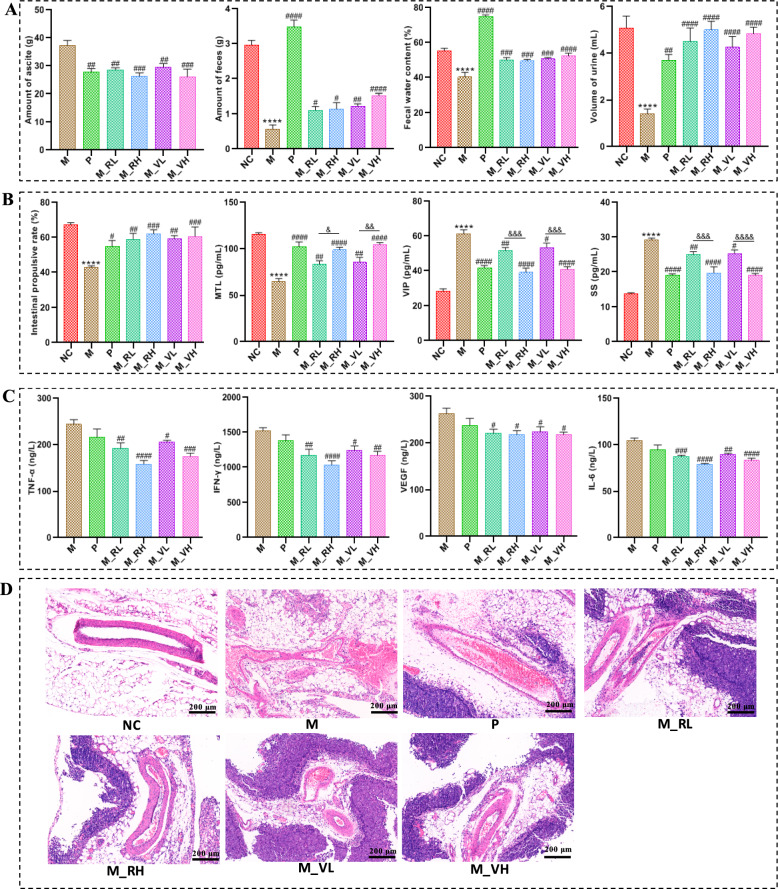
VSZT displayed comparable water-expelling effect to SZT in MAE rats (n = 6). **A** The amount of feces and ascite, fecal water content and urine volume of rats in all groups. **B** Intestinal propulsive rate and levels of related serum hormones of rats in all groups. **C** Levels of VEGF, IL-6, IFN-γ and TNF-α in ascites. **D** Representative morphology images of the mesenteric blood vessels. Compared with NC group, ****P < 0.0001; Compared with M group, ^#^*P* < 0.05; ^##^*P* < 0.01; ^###^*P* < 0.001; ^####^*P* < 0.0001

**Fig. 4:**
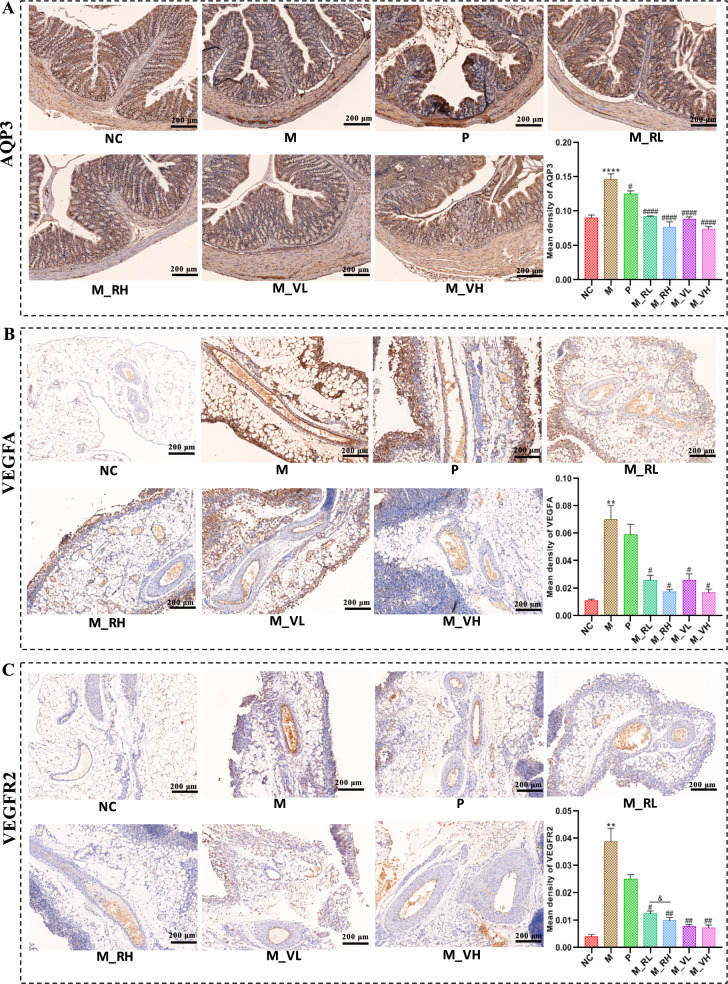
Both SZT and VSZT down-regulated expression levels of AQP3 in the colon as well as VEGFA and VEGFR2 in the mesenteric tissue (n = 6). **A** Representative immunohistochemical images and statistical analysis of expression levels of AQP3 in the colon. **B** Representative immunohistochemical images and statistical analysis of expression levels of VEGFA in the mesentery. **C** Representative immunohistochemical images and statistical analysis of expression levels of VEGFR2 in the mesentery. Compared with NC group, **P < 0.01; ****P < 0.0001; Compared with M group, ^#^*P* < 0.05; ^##^*P* < 0.01; ^####^*P* < 0.0001

As shown in Fig. 3A, obvious ascites formed in rats after modeling, and the volume of urine, amount of feces and fecal water content of rats in M group significantly decreased (*P* < 0.0001) compared with rats in NC group. After treated with magnesium sulfate or different doses of SZT/VSZT, all these indicators increased (*P* < 0.05) with reduced amount of ascites (*P* < 0.01) compared with M group. Then, intestinal motility associated indicators were detected, it can be seen from Fig. 3B that the intestinal propulsion rate and the levels of serum MTL decreased, while the levels of serum SS and VIP increased in rats of M group compared with NC group (*P* < 0.0001). After magnesium sulfate or different doses of SZT/VSZT treatment, all the indicators above were significantly restored (*P* < 0.05). Furthermore, since the formation of MAE is closely associated with inflammation, levels of VEGF, TNF-α, IL-1β and IFN-γ in the supernatant of ascites were detected (Fig. 3C). In comparison to M group, levels of them were significantly down-regulated after treatment with different doses of SZT/VSZT (*P* < 0.05). Besides, pathological damage in mesenteric vascular were analyzed and the results revealed that the vascular wall of the mesenteric vascular of rats in NC group was intact, with regular vascular morphology and normal thickness. For the rats in M group, the mesenteric neovascularization increased and the blood vessels congested. This might be due to tumor-induced damage to the blood vessel wall, resulting in partial defects and subsequent extravasation and edema. After administrated with different doses of SZT/VSZT, the congestion, edema, and extravasation of the mesenteric blood vessels of MAE rats were ameliorated (Fig. 3D). Given the documented correlations between AQP3 expression and fecal water content, as well as close connection between VEGFA/VEGFR2 expression and vascular permeability, we employed IHC method to comparatively evaluate the expression levels of AQP3 in the colon and VEGFA/VEGFR2 in the mesentery among groups (Fig. 4). The results revealed that the expression levels of all the proteins mentioned above showed a significant increase (*P* < 0.01) in the M group compared with NC group, and were significantly down-regulated (*P* < 0.05) by different doses of SZT/VSZT treatment.

Interestingly, for the indicators mentioned above, there was no significant difference on the restoration effect for the same indicator between SZT and VSZT at the same dose. These results indicated that SZT and VSZT exhibited comparable water-expelling efficacy in MAE rats, coupled with the fact that VSZT had lower intestinal toxicity than SZT as mentioned above, VSZT was further employed to explore the potential mechanisms underlying its action against MAE through integrative microbiomics and metabolomics analysis.

### VSZT improved serum endogenous metabolic disorders in MAE rats

**Fig. 5:**
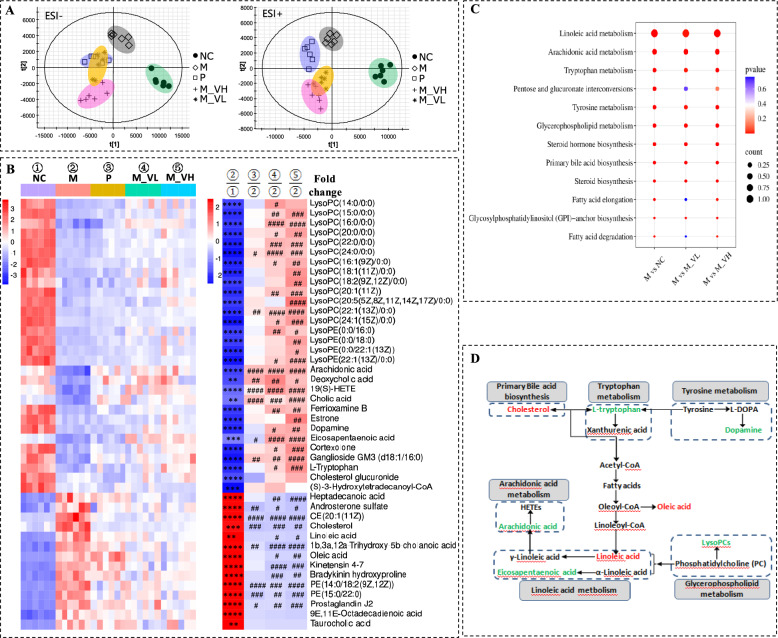
VSZT improved serum endogenous metabolic disorders in MAE rats (n = 6). **A** PLS-DA score plots of serum endogenous metabolites in positive and negative ion modes. **B** Heatmap of serum differential metabolites. **C** Disturbed metabolic pathways in different groups. **D** Topological network analysis diagram of representative serum differential metabolites and relevant metabolic pathways (Red represents up-regulated metabolites and green represents down-regulated metabolites in the M group). Compared with NC group, **P < 0.01; ***P < 0.001; ****P < 0.0001; Compared with M group, ^#^*P* < 0.05; ^##^*P* < 0.01; ^###^*P* < 0.001; ^####^*P* < 0.0001

A LC–MS based metabolomics analysis was conducted to investigate alterations in serum metabolites with or without VSZT treatment in MAE rats. To obtain repeatable results, the method was validated (Table S4). For intra-day precision, inter-day precision and stability tests of the targeted ions, the RSDs of retention time (*t*_*R*_) were calculated to be 0–0.17%, 0.05%–0.20% and 0.06%–0.31%, respectively. The RSDs of *m/z* were 0. The RSDs of intensity were varied within 1.12%–5.82%, 1.22%–5.33% and 2.11%–5.78%, respectively. The results indicated that the developed method was precise and stable enough for the analysis of serum samples. Then, PLS-DA models were constructed to assess the general metabolic alterations. As depicted in Fig. 5A, a clear separation between M and NC group in both positive and negative ion mode was observed, indicating that the serum metabolic profiles of MAE rats were significantly altered (*R*^2^*Y* ≥ 0.94, *Q*^2^ ≥ 0.52). After treatment with different doses of VSZT, the score plots of these groups approaching those of NC group.

A total of 44 serum metabolites that significantly changed in M group compared with NC group were selected as biomarkers, of which 30 were significantly decreased (*P* < 0.01) and 14 markedly increased (*P* < 0.01) (Fig. 5B and Table S5). Low and high dose of VSZT treatment significantly reversed 34 and 39 metabolites (*P* < 0.05), with 22 and 27 being significantly up-regulated and 12 and 12 being markedly down-regulated, respectively.

Further pathway analysis indicated that 11 pathways including linoleic acid metabolism, arachidonic acid metabolism, tryptophan metabolism, pentose and glucuronate interconversions, tyrosine metabolism, glycerophospholipid metabolism, steroid hormone biosynthesis, primary bile acid biosynthesis, steroid biosynthesis, fatty acid elongation, glycosylphosphatidylinositol (GPI)-anchor biosynthesis and fatty acid degradation were markedly altered in M group in comparison to NC group (*P* < 0.05). Low dose of VSZT treatment could significantly restore disordered pathways other than fatty acid elongation, pentose and glucuronate interconversions and fatty acid degradation (*P* < 0.05). High dose of VSZT treatment could significantly restore disordered pathways other than pentose and glucuronate interconversions (*P* < 0.05) (Fig. 5C). Moreover, to better understand the correlation between differential metabolites and disordered pathways, the topological network of representative metabolites and their related disordered pathways was mapped (Fig. 5D). These results indicated that VSZT treatment could partially restore MAE induced serum metabolic disorders.

### VSZT improved urinary endogenous metabolic disorders in MAE rats

Similarly, the method was validated and the results were showed in Table S6. For intra-day precision, inter-day precision and stability tests, it was found that the RSDs of *t*_*R*_ were calculated to be 0–0.19%, 0%–0.26% and 0%–0.24%, respectively; the RSDs of *m/z* were 0; the RSDs of intensity were varied within 0.84%–3.52%, 1.24%–10.68% and 1.63%–8.35%, respectively. The results indicated that the developed method was precise and stable enough for the analysis of urine samples.

**Fig. 6:**
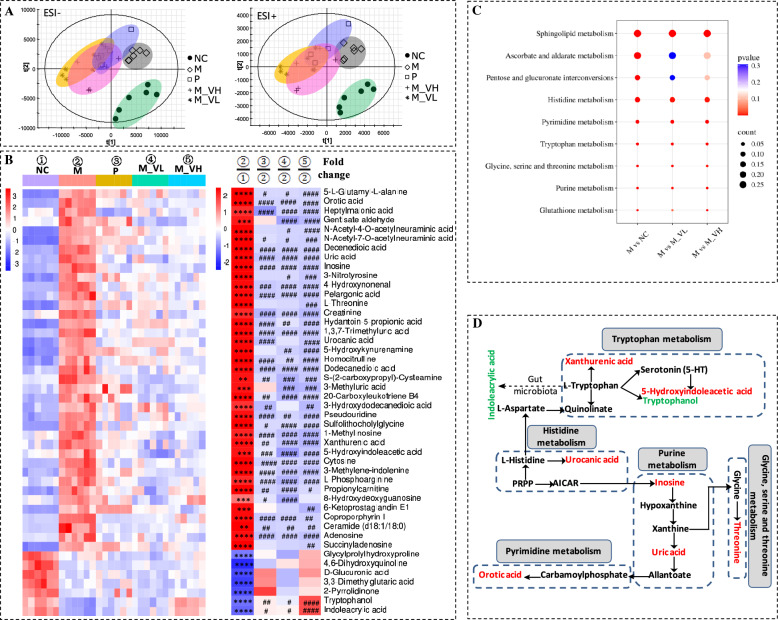
VSZT improved urinary endogenous metabolic disorders in MAE rats (n = 6). **A** PLS-DA score plots of urinary endogenous metabolites in positive and negative ion modes. **B** Heatmap of urinary differential metabolites. **C** Disturbed metabolic pathways in different groups. **D** Topological network analysis diagram of representative urinary differential metabolites and relevant metabolic pathways (Red represents up-regulated metabolites and green represents down-regulated metabolites in the M group). Compared with NC group, **P < 0.01; ***P < 0.001; ****P < 0.0001; Compared with M group, ^#^*P* < 0.05; ^##^*P* < 0.01; ^###^*P* < 0.001; ^####^*P* < 0.0001

PLS-DA analysis showed a clear distinction among the presented groups, particularly between M and NC group in both positive and negative ion mode, indicating that the urinary metabolic profiles of MAE rats were significantly interfered (*R*^2^*Y* ≥ 0.82, *Q*^2^ ≥ 0.52) (Fig. 6A). After treatment with different doses of VSZT, PLS-DA score plots of these groups approaching to those of NC group.

In comparison to NC group, totally 46 urinary metabolites that significantly changed in M group were selected as biomarkers, specifically 39 ones were remarkably up-regulated (*P* < 0.01) and 7 ones were remarkably down-regulated (*P* < 0.0001). As shown in Fig. 6B and Table S7, compared to M group, 36 and 40 out of 46 biomarkers were significantly reversed in M_VL and M_VH groups (*P* < 0.05), of which 34 and 38 were significantly down-regulated and 2 and 2 was markedly up-regulated, respectively.

Further pathway analysis showed that metabolites related to MAE were enriched in sphingolipid metabolism, ascorbate and aldarate metabolism, pentose and glucuronate interconversions, histidine metabolism, pyrimidine metabolism, tryptophan metabolism, glycine, serine and threonine metabolism, purine metabolism and glutathione metabolism (*P* < 0.05). Except for ascorbate and aldarate metabolism and pentose and glucuronate interconversions, all the disturbed pathways attributed to MAE could be restored by different doses of VSZT treatment (*P* < 0.05) (Fig. 6C). Moreover, to better understand the correlation between differential metabolites and disordered pathways, the topological network of representative metabolites and their related disordered pathways was mapped (Fig. 6D). These results indicated that VSZT treatment could partially restore MAE induced urinary metabolic disorders.

### VSZT reversed gut microbiota dysbiosis in MAE rats

**Fig. 7:**
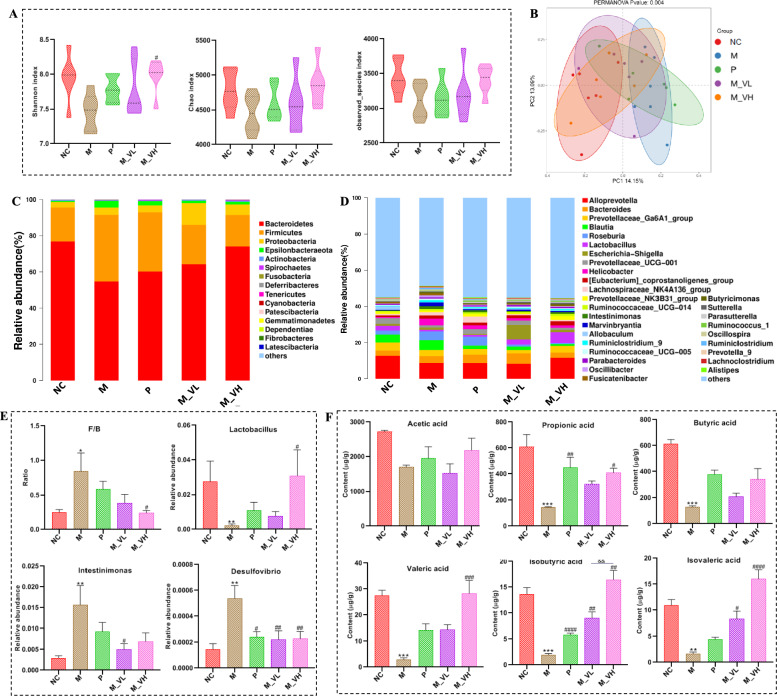
VSZT improved the disturbed gut microbiota and up-regulated the fecal SCFA’s decline in MAE rats (n = 6). **A** The *α*-diversity analysis of gut microbiota. **B** The PCoA score plots of *β*-diversity analysis for gut microbiota. **C, D** The relative abundance of the gut microbial composition at the phylum and genus levels. **E** The relative abundance of differential bacterial genera. **F** The contents of fecal SCFAs. Compared with NC group, *P < 0.05; **P < 0.01; ***P < 0.001; Compared with M group, ^#^*P* < 0.05; ^##^*P* < 0.01; ^###^*P* < 0.001; ^####^*P* < 0.0001

In order to explore the effect of VSZT on the gut microbiota of MAE rats, 16S rRNA sequencing was conducted. As shown in Fig. S1, the Specaccum accumulation curve and Rank abundance curve both tends to be flat, indicating that the amount of sequencing data was reasonable enough for further analysis. Estimation of *α*-diversity in terms of Shannon, Chao1 and Observed species index found that all the three indices were lower in M group in comparison to NC group, signifying a reduction in the richness and evenness of gut microbiota after MAE modeling. After treatment with different doses of VSZT, all the three indices could be partially recovered (Fig. 7A). Principal coordinate analysis (PCoA), estimation of *β*-diversity of the gut microbiota, revealed two distinct clusters between the inter-population microbial communities of the NC and M groups. Compared with M group, the distribution of each administration group shifted toward to NC group (Fig. 7B).

Then, differences in gut microbiota taxa and their relative abundance at the phylum and genus levels were further analyzed. As shown in Fig. 7C–E, at the phylum level, MAE rats had significant higher F/B (ratio of *Firmicutes* to *Bacteroidota*) values compared with the rats in NC group (*P* < 0.05), and the F/B values decreased significantly after treatment with high dose of VSZT (*P* < 0.05). At the genus level, compared with NC group, the M group exhibited a significantly increased relative abundance of *Desulfovibrio* (*P* < 0.01) and *Intestinimonas* (*P* < 0.01), along with a significantly decreased relative abundance of *Lactobacillus* (*P* < 0.01). Following treatment with low dose of VSZT, the relative abundance of *Intestinimonas* and *Desulfovibrio* were significantly restored (*P* < 0.05). In contrast, high dose of VSZT treatment led to a significant restoration in the relative abundance of *Lactobacillus* and *Desulfovibrio* (*P* < 0.05).

Moreover, because in the microecological environment, gut microbiota do not exist as isolated individuals, instead, they form a complex microecological co-occurrence network through direct or indirect interactions. Therefore, the interactions of microbial communities in NC, M, M_VL and M_VH groups at the genus level were analyzed. As shown in Fig. S2, the complexity of the bacterial networks of M_VL group was significantly greater than other groups, indicating more complex interaction patterns in this group than other ones. However, the complexity of other groups was similar (Table S8). To further determine the key genus of each group, ZiPi analysis was conducted. As shown in Fig. S3, 3, 2, 5 and 0 nodes fell in the module hubs, and 14, 13, 9 and 16 nodes fell in the connectors for NC, M, M_VL and M_VH group, respectively. Notably, not all the inter-group differential bacteria genus we focused on corresponded to the key nodes within intra-group. Interestingly, *Intestinimonas* corresponding to one of the key node in M group were also the key up-regulated bacterial genus of M group which could be restored by VSZT administration.

**Fig. 8:**
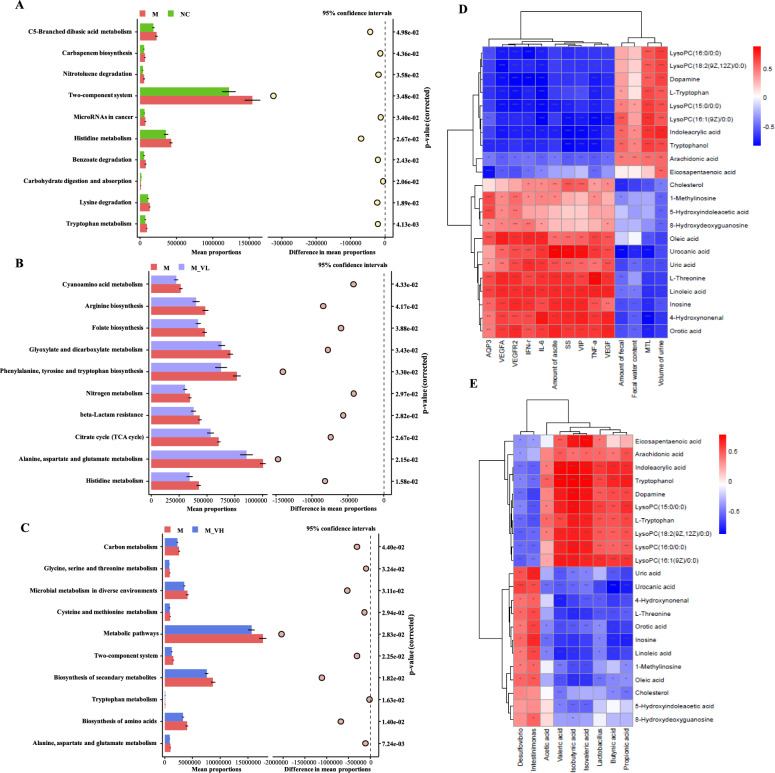
Functional KEGG pathways predicted by PICRUSt2 analysis and Spearman correlation analysis (n = 6). **A** Functional KEGG pathways between NC versus M. **B** Functional KEGG pathways between M versus M_VL. **C** Functional KEGG pathways between M versus M_VH. **D** Spearman correlation analysis between differential metabolites and effect-related indices. **E** Spearman correlation analysis between differential metabolites and gut microbiota. *P < 0.05; **P < 0.01; ***P < 0.001; ****P < 0.0001

After exploring the differences in microbiota composition among groups of MAE rats with or without VSZT administration, PICRUSt2 analysis was performed to predict differential level 3 KEGG pathways (Fig. 8A–C). Inter-group difference analysis revealed that several pathways, including tryptophan metabolism and histidine metabolism etc., were significantly up-regulated in M group compared with NC group (*P* < 0.05). Consistent with the findings of the metabolomics studies, this analysis indicated that low dose of VSZT intervention significantly down-regulated histidine metabolism (*P* < 0.05), whereas high dose of VSZT intervention notably down-regulated tryptophan metabolism in MAE rats (*P* < 0.05). All these results indicated that VSZT treatment could partially recover the disordered gut microbiota in MAE rats.

### VSZT partially restored the contents of fecal SCFAs in MAE rats

As shown in Fig. 7F, the contents of propionic acid, butyric acid, valeric acid, isobutyric acid and isovaleric acid in the M group were significant lower than that in the NC group (*P* < 0.01). Notably, except for acetic acid and butyric acid, the content of other SCFAs could be significantly restored by high dose of VSZT treatment (*P* < 0.05). These results indicated that VSZT treatment could partially restore MAE induced alterations of fecal SCFAs.

### Correlation analysis between differential metabolites and efficacy-related indicators, as well as between differential metabolites and gut microbiota

Aiming to explore the potential underlying mechanisms of VSZT against MAE, spearman correlations between differential metabolites and effect-related indices, as well as between differential metabolites and gut microbiota were conducted. As shown in Fig. 8D–E, fecal water content had positive correlations with tryptophanol and indoleacrylic acid (*P* < 0.05), while amount of ascites and AQP3 had negative correlations with L-tryptophan, tryptophanol and indoleacrylic acid (*P* < 0.001)*.* A positive association was observed between L-tryptophan, tryptophanol, indoleacrylic acid and *Lactobacillus* (*P* < 0.01). L-tryptophan, tryptophanol and indoleacrylic acid were metabolites of tryptophan metabolism pathway. Amount of ascites, VEGFA and VEGFR2 were negatively correlated with dopamine (*P* < 0.001), while dopamine was positively correlated with *Lactobacillus* (*P* < 0.01). Dopamine was metabolite of tyrosine metabolism pathway. The results indicated that VSZT alleviated MAE by regulating the abundance of *Lactobacillus*, tryptophan metabolism pathway, tyrosine metabolism pathway and modulating the expression of proteins like AQP3, VEGFA and VEGFR2.

### VSZT alleviated MAE by affecting cAMP-PKA-CREB-AQP3 signaling pathway

**Fig. 9:**
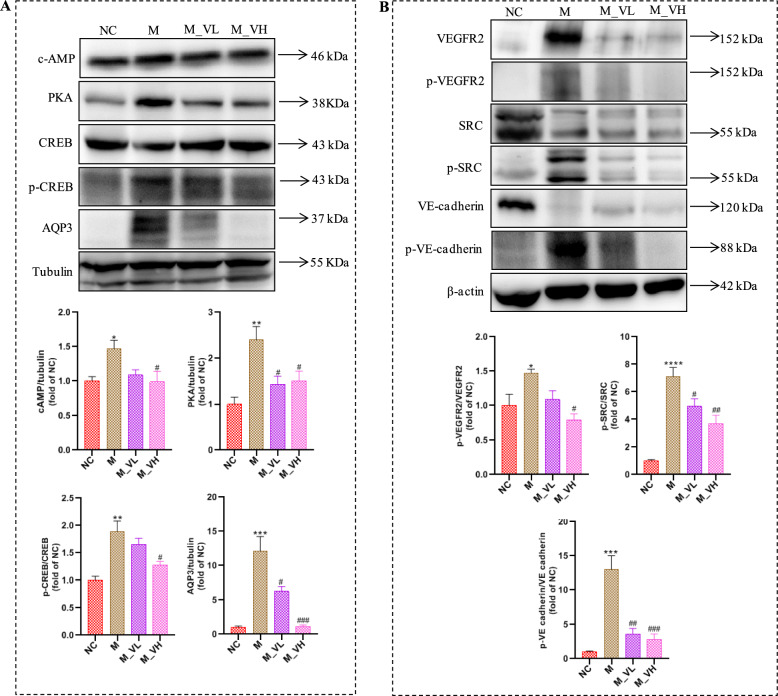
VSZT might alleviate MAE by affecting cAMP-PKA-CREB-AQP3 and VEGFA-VEGFR2-SRC-VE-cadherin signaling pathways (n = 3). **A** Western blotting analysis of cAMP, PKA, CREB, p-CREB and AQP3 in the colon. **B** Western blotting analysis of VEGFR2, p-VEGFR2, SRC, p-SRC, VE-cadherin and p-VE-cadherin in the mesentery. Compared with NC group, *P < 0.05; **P < 0.01; ***P < 0.001; ****P < 0.0001; Compared with M group, ^#^*P* < 0.05; ^##^*P* < 0.01; ^###^*P* < 0.001

As mentioned above, we found a potential link between the role of VSZT against MAE and the AQP3 related pathway. Further western blotting analysis was conducted to analyze the main proteins and the results found that, compared with NC group, the expression levels of cAMP, PKA, and AQP3, as well as relative protein ratio of p-CREB/CREB, were significantly increased in M group (*P* < 0.05). Notably, high dose of VSZT treatment could down-regulate the expression of these proteins and protein ratio (*P* < 0.05) (Fig. 9A). The results indicated that VSZT might display alleviation effect against MAE via affecting cAMP-PKA-CREB-AQP3 signaling pathway.

### VSZT alleviated MAE by affecting VEGFA-VEGFR2-SRC-VE-cadherin signaling pathway

As mentioned above, it was found that the role of VSZT against MAE was correlated with the VEGFA-VEGFR2 related pathway. Then, main proteins of VEGFA-VEGFR2 related pathway were analyzed and the results showed that, compared with NC group, the relative protein ratios of p-VEGFR2/VEGFR2, p-SRC/SRC, and p-VE-cadherin/VE-cadherin were significantly increased in M group (*P* < 0.05), while treatment with high dose of VSZT could down-regulate these aforementioned ratios (*P* < 0.05) (Fig. 9B). The results indicated that VSZT might display alleviation effect against MAE via affecting VEGFA-VEGFR2-SRC-VE-cadherin signaling pathway.

## Discussion

The clinical application of SZT is limited due to the presence of toxic herbs in its formula. Usually, as documented in Chinese Pharmacopoeia, for reducing toxicity, vinegar-processed KR and EPR are commonly used instead of raw ones clinically. However, whether VSZT, in which KR and EPR were substituted with their vinegar-processed counterparts, exhibits the effect of toxicity reduction with efficacy conservation in MAE rats, and the potential underlying mechanisms of VSZT against MAE, remain unclear. In the current study, a comparative investigation was conducted on the quality, toxicity and efficacy of SZT and VSZT, as well as the potential mechanisms thereof in MAE rats.

To comparatively investigate the quality of SZT and VSZT, main components of them were selected for quantitative analysis. Here, **6–9** are jatrophane-type diterpenes, while **4**, **10** and **13** belong to ingenane-type diterpenes. Of note, ingenane-type and jatrophane-type diterpenes derived from KR could cause irritation and inflammation [23]. Our previous studies indicated that **10** and **13** were more toxic than **4** in terms of intestinal toxicity [24, 25]. Interestingly, **13** could be converted to **4** under acidic conditions through the breakage of ester bond, and both of them possessed significant effect in treating MAE [26]. In addition, our previous studies also showed that triterpenoid components in KR with an 8-ene-7-one structure had strong cytotoxicity and could inhibit the proliferation of small intestinal crypt cells IEC-6 in a concentration-dependent manner [20], while **11** and **12** are such triterpenoids. Therefore, VSZT had lower content of more toxic **7**–**8** and **10**–**13** but higher content of less toxic **4** than SZT. The results suggested that VSZT might be less toxic than SZT. However, toxicity and efficacy of VSZT versus SZT requires further comparative exploration.

Our previous studies found that SZT exerted primarily intestinal toxicity and the pathological damage of VSZT was lower than that of SZT in normal rats [16, 18]. Besides, intestinal oxidative damage of SZT was found in MAE rats and FJ cooperated with other members in SZT could reduce intestinal injury [14]. However, whether the intestinal damage of VSZT was lower than that of SZT in MAE rats was unknown. Therefore, the intestinal damage of SZT and VSZT was comparatively studied in this study. Occludin, ZO-1, LPS and DAO are commonly used to evaluate intestinal barrier function, an important aspect used to evaluate intestinal damage [27, 28]. For example, LPS can bind to toll-like receptor 4 and activate NF-κB, thereby triggering intestinal inflammation, damaging intestinal barrier function, and increasing intestinal permeability [29]. In addition, oxidative damage may contribute to deterioration of intestinal barrier damage and its related indicators such as LDH, GSH and SOD were detected [30]. Our results found increased levels of ZO-1 and occludin in groups administered with VSZT compared with groups administered with SZT, and this might be owing to decreased levels of LPS and DAO, as well as alleviated oxidative damage. The results showed that VSZT showed lower intestinal injury than SZT in MAE rats.

MAE frequently causes gastrointestinal motility disorders like bloating and constipation [3], while elevated levels of SS or VIP or decreased levels of MTL signify weakened gastrointestinal motility [31]. Besides, AQP3 is primarily expressed in colon, where it plays crucial role in regulating water absorption from the luminal side to the vascular side of the colon, and thereby modulates fluid metabolism during the development of ascites [14, 32]. In addition, inflammation is necessary for the formation of MAE. The abnormally changed cytokines such as TNF-α and IL-6 in MAE can destroy tissue matrix, promote cancer cell metastasis, and induce the production of VEGF, a key factor crucial for MAE progression [33]. Multiple VEGFs (VEGF-A, VEGF-B, VEGF-C and VEGF-D) interact with VEGF receptors such as VEGFR1, VEGFR2 and VEGFR3. Among them, VEGFA and VEGFR2 is prominently involved in angiogenesis and vascular permeability, thereby affecting the accumulation of ascites [34–37]. Our study revealed that both SZT and VSZT treatment decreased the amount of ascites by increasing fecal water content which is similar to magnesium sulfate, promoting defecation and reducing vascular permeability. Promoted defecation was attributed to the increased intestinal propulsion rate and serum MTL levels as well as decreased serum SS and VIP levels. Increased fecal water content was related to the reduction of AQP3 expression levels in colon. Reduced vascular permeability was due to the declined levels of VEGF, TNF-α, IL-1β and IFN-γ in the supernatant of ascites and expression levels of VEGFA and VEGFR2 in mesenteric tissue. However, no obvious difference in efficacy in MAE rats between SZT and VSZT treatment was found, indicating the efficacy of VSZT had been retained compared to SZT. Therefore, the toxicity reduction with efficacy conservation of VSZT was confirmed and the underlying mechanisms of VSZT were explored.

MAE causes alterations in endogenous metabolism in patients, and this alteration is strongly associated with patient survival [38]. We previously found that efficacy of compositional herbs of VSZT against MAE was associated with modulation of the gut microbiota and tryptophan metabolism [13, 14]. Therefore, we preliminarily explored the underlying mechanisms by which VSZT alleviates MAE through integrated analysis of metabolomics and 16S rRNA analysis. Reduced systemic L-tryptophan had been found in patients with cancer, which would promote tumor progression by inhibiting antitumor immune responses. Tryptophan is a precursor of 5-hydroxytryptamine (5-HT), while 5-HT can be further metabolized to 5-Hydroxyindoleacetic acid (5-HIAA). Besides, tryptophan can also be degraded to tryptophanol, indoleacrylic acid and xanthurenic acid through different pathways [39, 40]. It was reported that 5-HT can activate protein Gs to stimulate adenylate cyclase, increasing cAMP to activate PKA. The up-regulated cAMP/PKA signaling promotes AQP3 phosphorylation and expression [41]. Tryptophanol can activate the aryl hydrocarbon receptor (AhR) and then induces the expression of IL-22 followed by regulating epithelial integrity and immunity. Indoleacrylic acid, a metabolite derived from bacterial metabolism of tryptophan, serves as a ligand for AhR. It exhibites beneficial effects on intestinal epithelial barrier function and alleviates the inflammatory response of immune cells [39, 42]. Reportedly, intestinal water metabolism is related to epithelial cells in the intestinal mechanical barrier [41]. In the present study, VSZT treatment increased L-tryptophan, tryptophanol and indoleacrylic acid levels, and decreased 5-HIAA level in MAE rats, indicating that VSZT might increase intestinal water metabolism and decrease inflammation by restoring tryptophan metabolism disorders and AQP3 related pathway. It was reported that dopamine could inhibit the phosphorylation of VEGFR2 in endothelial cells through dopamine D2 receptors, thereby regulating VEGF mediated vascular permeability and angiogenesis [43]. Our data showed that VSZT increased serum levels of dopamine and decreased content of VEGF in the supernatant of ascites in MAE rats, indicating that VSZT might decrease vascular permeability and angiogenesis by reversing tryrosine metabolism disorders and VEGF related pathway.

The disruption of gut microbiota homeostasis is closely related to the progression of MAE. Our previous studies found that FJ cooperated with other herbs in SZT to alleviate MAE relied on the gut microbiota [14]. Furthermore, it has been reported that when ascites occured, the homeostasis of gut microbiota was destroyed, leading to changes in the intestinal barrier, further leading to the release of inflammatory cytokines, which in turn leads to aggravation of ascites [5, 6]. Then, the gut microbiota and its metabolites SCFAs, important for intestinal homeostasis, were analyzed [44]. In current study, we revealed that high dose of VSZT reversed the elevated F/B, decreased relative abundance of *Lactobacillus* and increased relative abundance of *Desulfovibrio* and *Intestinimonas*, as well as declined contents of SCFAs in MAE rats. Reportedly, changes in the F/B, associated with inflammation and cancer, are considered the first sign of gut microbota imbalance [45]. *Lactobacillus* can reduce the mRNA expression of cytokines such as TNF-α, IFN-γ, IL-1β, and IL-6, thereby attenuating inflammation and lowering the incidence of tumorigenesis [46]. *Intestinimonas* was reported with higher abundance in a variety of inflammation related diseases and its abundance was positively correlated with various inflammatory factors like TNF-α [47, 48]. *Desulfovibrio* is an opportunistic pathogen and will grow excessively in the environment of various intestinal and extraintestinal diseases. Oral administration of this bacterium can cause a slowdown in the intestinal motility of mice. In addition, the massive proliferation of this bacterium can lead to the massive production of LPS, thereby causes the release of pro-inflammatory factors [29, 49, 50]. In addition, as important fuels for intestinal epithelial cells (IECs), SCFA can regulate IECs functions, thereby affecting intestinal motility, intestinal barrier function and host metabolism. For example, propionic acid, which could be produced by *Lactobacillus* [51], can activate GPR41 and then lead to the inhibition of cAMP accumulation and PKA activation. Reportedly, intestinal water metabolism could be regulated by AQP3 through cAMP/PKA pathway [41, 52]. Our results demonstrated that VSZT alleviates MAE by reducing inflammation and regulating intestinal water metabolism through reversing gut microbiota and SCFAs disorders.

**Fig. 10:**
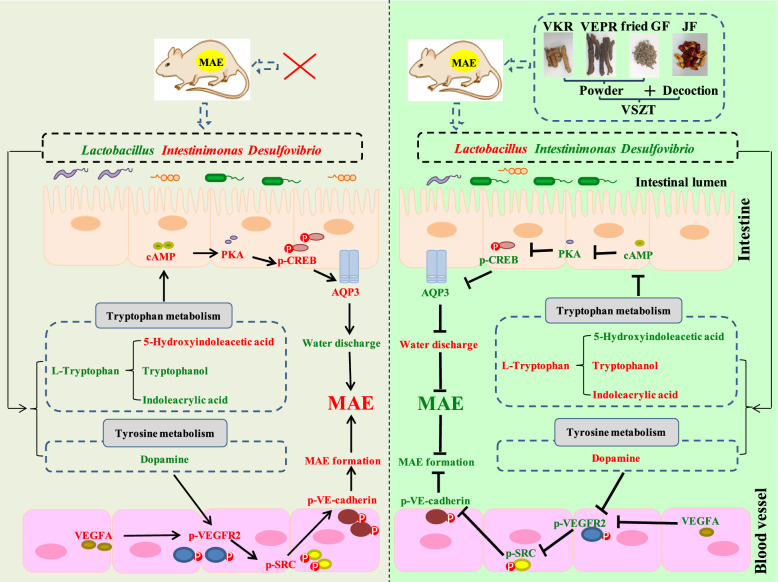
Schematic representation of VSZT increased water excretion and decreased MAE formation to alleviate MAE through regulating gut microbiota, restoring tryptophan and tyrosine metabolism disorders, and affecting cAMP-PKA-CREB-AQP3 and VEGFA-VEGFR2-SRC-VE-cadherine pathway. Red front: up-regulation; Green front: down-regulation; ↑: Activiation; ┤: Inhibition

Alterations in the composition and abundance of gut microbiota may influence the metabolism of tryptophan. Notably, *Lactobacillus*, as gut commensals, can produce indoles through tryptophan metabolism [39]. Our study revealed that tryptophan, tryptophanol and indoleacrylic acid were positively correlated with *Lactobacillus*, and these metabolites were important in the tryptophan metabolism pathway. Reportedly, tryptophan-derived indoles such as tryptophanol and indoleacrylic acid act as aryl hydrocarbon receptor (AhR) agonists and are important for epithelial renewal and barrier integrity [39]. Barrier dysfunction exacerbates inflammation and impairs the water-holding capacity of the gastrointestinal tract, disrupting intestinal absorption and metabolism of water and electrolytes, thereby affecting fecal water content [53]. Besides, our data indicated that AQP3 has negative association with tryptophan, tryptophanol and indoleacrylic acid, while has positive association with 5-HIAA. 5-HT, precursor of 5-HIAA, is produced from tryptophan and can regulate the expression of AQP3 through cAMP/PKA pathway, thereby regulating fecal water content [41]. Reportedly, cAMP can activate PKA, and activated PKA can phosphorylate CREB (p-CREB), while p-CREB can increase the expression levels of AQP3, thereby decreasing the water content in stool [32]. Our data indicated that VSZT could down-regulate the expression levels of cAMP and PKA, as well as protein ratio of p-CREB/CREB, followed by down-regulated expression levels of AQP3, suggesting that VSZT might regulate the metabolic levels of tryptophan metabolism by increasing the abundance of *Lactobacillus*, thereby promoting water excretion of MAE rats through affecting cAMP-PKA-CREB-AQP3 signaling pathway (Fig. 10). Furthermore, the present studies showed that dopamine, a metabolite of tyrosine metabolism, was positively correlated with *Lactobacillus*, while VEGFA and VEGFR2 were negatively correlated with dopamine. It was reported that *Lactobacillus* could express tyrosine decarboxylase and then convert levodopa to dopamine [54], while dopamine could inhibit the phosphorylation of VEGFR2 in endothelial cells in vivo through dopamine D2 receptors, thereby regulating vascular permeability mediated by VEGFA [43]. Reportedly, when VEGFA binds to VEGFR2, VEGFR2 is activated by phosphorylation at Tyr951 site. Activated VEGFR2 activates SRC by phosphorylation at Tyr416 site, and activated SRC acts on the Tyr685 site of VE-cadherin, resulting in SRC-dependent tyrosine phosphorylation of VE-cadherin, internalisation, and disassembly of adhesion junctions, leading to an increase in vascular endothelial permeability [36, 55, 56]. We found that VSZT could down-regulate the protein ratios of p-VEGFR2/VEGFR2, p-SRC/SRC and p-VE-cadherin/VE-cadherin, suggesting that VSZT might increased metabolic levels of tyrosine metabolism by increasing the abundance of *Lactobacillus*, thereby decreasing vascular permeability of MAE rats through affecting VEGFA-VEGFR2-SRC-VE-cadherin signaling pathway (Fig. 10).

In summary, this work comparatively studied the quality, toxicity and efficacy of SZT and VSZT, and explored the potential mechanisms of VSZT against MAE. Although there are limitations in the current study regarding lack of further in-depth verification, our findings are indeed encouraging, revealing that VSZT had significant therapeutic potential in treating MAE with lower intestinal toxicity, as well as the potential mechanisms of VSZT against MAE might be closely related to gut microbiota, tryptophan metabolism and tyrosine metabolism. Future research endeavors could employ microbiota transplantation and targeted metabolomics to validate the mediating role of gut microbiota in tryptophan metabolism and tyrosine metabolism, or employ silencing or overexpression techniques to validate the role of AQP3 and VEGFA-VEGFR2 related signaling pathway in VSZT’s treatment of MAE.

## Conclusions

In conclusion, VSZT preserve the efficacy of SZT on MAE with lower intestinal toxicity. Furthermore, by integrating metabolomics, 16S rRNA and Western blotting analysis, we found that VSZT remodels the gut microbiota in MAE rats, especially elevating the relative abundance of *Lactobacillus.* On the one hand, this restructuring might reverse the disordered tryptophan metabolism, thereby promoting water excretion of MAE rats through affecting cAMP-PKA-CREB-AQP3 signaling pathway. On the other hand, elevated relative abundance of *Lactobacillus* might promote tyrosine metabolism, which in turn reduced vascular permeability by affecting VEGFA-VEGFR2-SRC-VE-cadherin signaling pathway and subsequent reduced MAE formation. This paper will provide a scientific basis for the safe and effective use of VSZT in clinical practice.

## Supplementary Information

Additional file1

Additional file2

Additional file 3. **Fig. S1** Specaccum curve and Rank abundance curve of gut microbiota analysis (*n* = 6). (**A**) Specaccum curve. (**B**) Rank abundance curve. **Fig. S2** Co-occurrence network analysis of microbial interactions at the genus level of NC, M, M_VL and M_VH group. The size of the node is directly proportional to the connectivity of the OUT. The red edge represents positive correlation, and the green edge represents negative correlation. **Fig. S3** Plots of ZiPi analysis for bacteria in NC, M, M_VL and M_VH group (*n* = 6).

## Data Availability

The data associated with this study can be obtained from the corresponding author upon reasonable request.

## Abbreviations

SZT: Shi–Zao–Tang
VSZT: SZT containing vinegar-processed KR and EPR
MAE: Malignant ascites effusion
KR: Kansui Radix
VKR: Vinegar-processed KR
EPR: Euphorbiae Pekinensis Radix
VEPR: Vinegar-processed EPR
GF: Genkwa Flos
JF: Jujubae Fructus
UPLC-TQ-MS: Ultra high performance liquid chromatographic coupled with triple quadrupole tandem mass spectrometry
UHPLC-QTOF-MS: Ultra-performance liquid chromatography coupled with quadrupole time-of-flight mass spectrometry
GC–MS: Gas chromatography-mass spectrometry
WB: Western blotting
H&E: Hematoxylin eosin staining
IHC: Immunohistochemistry
ELISA: Enzyme-Linked Immunosorbent Assay
SCFAs: Short chain fatty acids
LDH: Lactate dehydrogenase
GSH: Glutathione
SOD: Superoxide Dismutase
PCoA: Principal coordinates analysis
PLS-DA: Partial least squares discriminant analysis
OPLS-DA: Orthogonal partial least squares discriminant analysis
VEGF: Vascular endothelial growth factor
IFN-γ: Interferon-γ
IL-6: Interleukin-6
TNF-α: Tumor Necrosis Factor-α
LPS: Lipopolysaccharide
DAO: Diamine oxidase
ZO-1: Zona Occludens 1
MTL: Motilin
VIP: Vasoactive intestinal peptide
SS: Somatostatin
AQP: Aquaporin
VIP: Variable important in projection
FC: Fold change
16S rRNA: 16S ribosomal RNA gene
VEGFA: Vascular endothelial growth factor A
VEGFR2: Vascular endothelial growth factor receptor 2
SRC: SRC protein
VE-cadherin: Vascular endothelial cadherin
cAMP: Cyclic adenosine monophosphate
PKA: Protein kinase A
CREB: CAMP response element-binding protein
