# Supplementary material for: Feasibility of vinegar processing of toxic herbs in Shi–Zao–Tang: toxicity reduction, efficacy preservation in malignant ascites rats and underlying pharmacodynamic mechanisms
Source: Chin Med. 2025 Oct 4;20:156. doi: 10.1186/s13020-025-01224-9 (PMC12495871; doi:10.1186/s13020-025-01224-9)
Supplement: Supplementary file 1 — Additional file1 [file 13020_2025_1224_MOESM1_ESM.docx]

**Supplementary materials**

***Chemicals and reagents***

Glutathione (GSH, Lot 20220519), Lactate dehydrogenase (LDH, Lot 20220519) and Superoxide dismutase (SOD, Lot 20220519) detection kits were all purchased from Nanjing Jiancheng Institute of Biological Engineering (Nanjing, China). Rat TNF-α (Lot 20220518-RX302058R), IL-6 (Lot 20220518-RX302856R), IFN-γ (Lot 20220518-RX302900R) and VEGF (Lot 20220511-RX302205R) enzyme linked immunosorbent assay (ELISA) kits were purchased from Quanzhou Ruixin Biological Technology Co., Ltd (Quanzhou,China). Rat Lipopolysaccharide (LPS, Lot 20220617003), Diamine oxidase (DAO, Lot 20220617003), Motilin (MTL, Lot 20220618003), Vasoactive intestinal peptide (VIP, Lot 20220618003) and Somatostatin (SS, Lot 20220618003) ELISA kits were purchased from Nanjing MALLBIO Biological Technology Co., Ltd (Nanjing, China). BCA kits (Lot 092721220323) was purchased from Beyotime Biotech Inc, (Shanghai, China).

All primary antibodies are rabbit antibodies. Antibodies for immunohistochemical staining such as anti-AQP3 (Lot bs-4611R) antibody was obtained from Beijing Biosynthesis Biotechnology Co., Ltd. (Beijing, China), anti-VEGFA (Lot #83m8093) and anti-VEGFR2 (Lot #7897989) antibody were purchased from Affinity Biosciences LTD, anti-ZO-1 (Lot #44h7470) and anti-Occludin (Lot #53t8241) antibody were purchased from Affinity Biosciences Ltd (Changzhou, China). Antibodies for western blotting such as anti-Phospho-VEGFR2 (Tyr951) (Lot #20x3863) and anti-VE-cadherin (Lot #3412780) antibody were purchased from Affinity Biosciences Ltd (Changzhou, China), anti-SRC, anti-PKA (Lot 00042029) and *β*-actin (Lot 00112007) were purchased from ProteinTech Inc (Wuhan, China), anti-VEGFR2 (Lot GR3414304-6), anti-VE Cadherin (phosphor Y685) (Lot GR3295417-20), anti-cAMP (Lot 1001732-4) protein kinase catalytic subunit and anti-CREB (phosphor S133) (Lot 1007881-3) antibody were purchased from Abcam (Cambridge, UK), anti-Phospho-Src (Tyr416) (Lot 10) and anti-CREB (Lot 20) antibody were purchased from Cell Signaling Technology (Danvers, MA, USA), anti-AQP3 (Lot 383A3A11) antibody were obtained from Thermo Fisher Scientific (Waltham, MA), anti-*β*-tubulin (Lot 3507438006) antibody was obtained from ABclonal Technology Co., Ltd (Wuhan, China). HPR conjugated affinipure goat anti-rabbit Ig G (H+L) (Lot BST17E13B17F54) was obtained from Boster Biological Technology Co., Ltd. (Wuhan, China). ECL detection reagents were obtained from Dalian Meilun Biotechnology Co., Ltd (Dalian, China).

**Supplementary methods**

***Comparative assessment of the quality of SZT and VSZT using UPLC-TQ-MS/MS analysis***

LC conditions: UPLC analysis was performed on a Waters ACQUITY UPLC^TM^ system (Waters corporation, Milford, MA, USA), which was equipped with a binary solvent delivery manager and auto-sampler. Chromatographic separation was achieved on a Waters ACQUITY HSS T3 C18 column (2.1 mm×100 mm, 1.8 µm). A mobile phase consisting of 0.1% formic acid in water (A) and 0.1% formic acid in acetonitrile (B) was applied with the optimized gradient program as follows: 0-1 min, 35% B; 1-7 min, 35%-100% B; 7-15.5 min, 100% B; 15.5-16 min, 100%-35% B; 16-18 min, 35% B. The flow rate was 0.4 mL/min. The column and auto-sampler temperature were maintained at 40ºC and 10ºC, respectively. The injection volume was 2.0 µL.

Mass spectrometry: Detection and quantification was performed on a Waters Xevo TQ-S Micro Mass Spectrometer with electrospray ionization (ESI) interface in multiple reaction monitoring mode (MRM) under positive and negative acquisition mode. The capillary voltage was 2.5 kV. The source temperature was 150ºC. The desolvation temperature was 500ºC. The desolvation gas flow rate was 800 L/h. The mass parameters including mass transitions, cone and collision energy for targeted compounds and IS were automatically optimized with the IntelliStart procedures of the Masslynx V4.2 software. The corresponding specific ion pairs for monitoring, cone voltage, collision energy and time range of each analyte were summarized in Table S1.

Method validation: The method for quantitative analysis of main components was validated in terms of linearity, sensitivity, precision, repeatability, stability, accuracy and matrix effect. The mixed stock solutions of the reference compounds were diluted to a series of concentrations for construction of calibration curves. The calibration curves were determined by plotting the peak area ratios of the analytes to IS (y) versus the concentration of the analytes (x). The equation was further optimized by least-square weighted (1/x^2^) linear regression analysis. The limit of detection (LOD) and quantification (LOQ) were expressed as the concentration of each analyte at a signal-to-noise (S/N) ratio of 3 and 10, respectively. The precision was determined by analyzing the same sample for six replicates within one day. To evaluate the repeatability, six SZT samples were prepared in parallel and analyzed. The stability test was performed by determining the sample solution during the duration of 0, 1, 2, 4, 6, 8, 10, 12, 18 and 24 h, respectively. Accuracy of the assay was evaluated by the spike recovery test. Known amount of the mixed reference compounds (the amount of each reference compound equivalent to 100% of original content) was spiked into SZT samples, and then the samples were extracted and analyzed. The spike recoveries were calculated by following equation: spike recovery (%) = (mean detected amount - original amount) / spiked amount × 100%. All the variations in precision, repeatability, stability and accuracy were expressed as relative standard deviation (RSD). The matrix effect was studied by comparing the slopes of the calibration standard curves with the standards prepared in the matrix with the curves slope of the standards prepared in the solvent.

***Metabolomics analysis***

Analytical conditions: Chromatographic separation was performed on a Waters ACQUITY UPLC^TM^ I class system (Waters corporation, Milford, MA, USA) with a ACQUITY UPLC HSS T3 column (2.1×100 mm, 1.8 µm). The mobile phase was consisted of water (A) and methanol (B), both containing 0.1% formic acid (v/v). The serum samples were eluted with the gradient elution as follows: 0-1 min, 40%B; 1-4 min, 40%-80%B; 4-10 min, 80%-100%B; 10-14 min, 100%B; 14-15 min, 100%-40%B; 15-17 min, 40%B. The urine samples were eluted with the gradient elution as follows: 0-1 min, 5%B; 1-10 min, 5%-100%; 10-14 min, 100%B; 14-15 min, 100%-5%B; 15-17 min, 5%B. The flow rate was 0.3 mL/min. The column and auto-sampler were maintained at 40 and 10ºC, respectively. The injection volume was 2 µL. Mass detection was performed on a Waters Synapt G2-S Q-TOF mass spectrometer (Waters corporation, Milford, MA, USA) equipped with electrospray ionization (ESI) interface. The operation parameters were set as follows: capillary voltage, 2.5 kV; source temperature, 120ºC; desolvation temperature, 450ºC; desolvation gas flow rate, 800 L/h; cone voltage, 40 V; cone gas flow rate, 50 L/h. Continuum data were acquired for each sample range from 50 to 1500 Da. The low and high collision energy was set at 6 V and 20-40 V, respectively.

Method validation: According to reference [1], peak retention time, signal intensity and mass accuracy of ten ions (*m/z* 616.1727, *m/z* 496.3483, *m/z* 524.3760, *m/z* 360.3347 and *m/z* 725.5530 in positive mode and *m/z* 512.2750, *m/z* 514.2936, *m/z* 540.3384, *m/z* 568.3688 and *m/z* 747.5739 in negative mode in serum samples; *m/z* 188.9891, *m/z* 212.0106, *m/z* 178.0560, *m/z* 201.0275 and *m/z* 343.0897 in negative ion mode and *m/z* 245.1639, *m/z* 397.0688, *m/z* 321.0975, *m/z* 398.2441 and *m/z* 360.3264 in positive ion mode in urine samples) of the major peaks distributed in the chromatograms of QC sample with different retention times were selected for the method validation. The method was validated in terms of intra-day precision, inter-day precision and stability. For intra-day precision test, QC solution was analyzed in six times within one day, while for inter-day precision tests, the same sample was analyzed in triplicates for consecutive two days. The QC solution was analyzed at 0, 2, 4, 6, 8, 12, 14, 16, 24 and 36 h to determine the stability of the assay. The relative standard deviations (RSDs) were used to evaluate the precision and stability.

***Determination of short chain fatty acids (SCFAs)***

According to our previous study [2], the contents of SCFAs were determined in feces. Briefly, 200 mg of feces was weighted into a tube and 1 mL of distilled water was added into it followed by homogenizing at 70 Hz for 180 s. Then, the homogenate was centrifuged at 13000 rpm at 4ºC for 10 min. 500 µL of supernatant was collected and transferred into a tube, added with 20 µL of internal standard (2-methylvaleric acid, 1.0 µL/mL) and 50 µL of 50% HCl, vortexed for 20 s, added with 570 µL of diethyl ether, shook at 70 Hz for 3 min, centrifuged at 13000 rpm at 4ºC for 10 min, and the upper layer liquid was collected for the GC-MS analysis.

Sample analysis was performed on a 7890B gas chromatograph system coupled to a 7000D mass spectrometer (Agilent Technologies, Santa Clara, CA, USA). The chromatographic separation was carried out on an Agilent J&W DB-FFAP column (15 m×0.25 mm×0.25 μm) with an injection volume of 1 µL and splitting ratio of 10:1. The Helium was used as carrier gas at the flow rate of 1.5 mL/min. The injection port temperature was set at 230ºC. The optimal column oven temperature program was set as follow: 0-3 min, 80-140ºC; 3-4 min, 140-150ºC; 4-5 min, 150ºC; 5-7 min, 150-180ºC; 7-8 min, 180-200ºC; 8-9 min, 200-230ºC; and finally kept at 230ºC for 2 min. The solvent delay was set at 3 min. Detection and quantification was performed on multiple reaction monitoring mode (MRM) under positive acquisition mode with an electron impact ion source. The ionization voltage was 70 eV. Temperature of ion source, quadrupole and interface was 230, 150 and 250ºC, respectively. The optimal precursor ion, product ion and collision energy of each SCFAs were shown in Table S9.

**References**

1. Zhou L, Xu J-D, Zhou S-S, Zhu H, Kong M, Shen H, et al. Independent or integrative processing approach of metabolite datasets from different biospecimens potentially affects metabolic pathway recognition in metabolomics. J Chromatogr A. 2019; 1587: 146-154.

2. Zhu J-H, Mao Q, Wang S-Y, Liu H, Zhou S-S, Zhang W, et al. Optimization and validation of direct gas chromatography-mass spectrometry method for simultaneous quantification of ten short-chain fatty acids in rat feces. J Chromatogr A. 2022; 1669: 462958.
