# Supplementary material for: Feasibility of vinegar processing of toxic herbs in Shi–Zao–Tang: toxicity reduction, efficacy preservation in malignant ascites rats and underlying pharmacodynamic mechanisms
Source: Chin Med. 2025 Oct 4;20:156. doi: 10.1186/s13020-025-01224-9 (PMC12495871; doi:10.1186/s13020-025-01224-9)
Supplement: Supplementary file 2 — Additional file2 [file 13020_2025_1224_MOESM2_ESM.docx]

**Table S1** MRM parameters of 14 target quantitative compounds and internal standard in SZT and VSZT

| No. | Analyte | RT (min) | Ion mode | MRM Transitions (Parent→Daughter) | Cone (V) | Collision Energy (eV) | Time  range (min) |
| --- | --- | --- | --- | --- | --- | --- | --- |
| 1 | Luteolin | 1.66 | ES+ | 287.03→152.97 | 30 | 31 | 1.0-4.0 |
| 2 | Quercetin | 1.72 | ES+ | 303.25→136.99 | 31 | 30 | 1.0-4.0 |
| 3 | Kaempferol | 2.73 | ES+ | 287.03→152.97 | 30 | 31 | 1.0-4.0 |
| 4 | Ingenol | 2.63 | ES- | 347.14→329.18 | 40 | 6 | 1.0-4.0 |
| 5 | Genkwanin | 4.29 | ES+ | 285.16→167.03 | 60 | 28 | 3.5-5.5 |
| 6 | Kansuinin C | 5.26 | ES- | 767.28→599.36 | 10 | 13 | 4.5-6.5 |
| 7 | Kansuinin B | 5.37 | ES- | 767.28→599.36 | 10 | 13 | 4.5-6.5 |
| 8 | Kansuinin A | 5.85 | ES+ | 731.16→671.08 | 40 | 8 | 4.5-6.5 |
| 9 | Kansuinin E | 6.57 | ES+ | 778.33→124.03 | 52 | 42 | 5.5-7.5 |
| 10 | Kansuiphorin C | 7.20 | ES+ | 479.31→105.01 | 24 | 28 | 6.5-8.5 |
| 11 | Eupha-8,24-diene-3*β*,11*β*-diol-7-one | 7.44 | ES+ | 457.33→251.17 | 52 | 18 | 6.5-8.5 |
| 12 | Tirucalla-8,24-diene-3*β*,11*β*-diol-7-one | 7.61 | ES+ | 457.33→251.17 | 52 | 18 | 6.5-8.5 |
| 13 | 3-*O*-(2’*E*,4’*Z*-decadienoyl)-20-*O*-acetylingenol | 8.04 | ES+ | 541.15→313.15 | 20 | 12 | 7.7-8.5 |
| 14 | Euphol | 14.91 | ES+ | 409.41→191.17 | 10 | 10 | 13.0-17.0 |
| IS | Loratadin | 3.52 | ES+ | 383.11→337.09 | 50 | 24 | 3.0-4.5 |

**Table S2** The results of methodological validation

| No. | Linearity | | | Sensitivity (ng/mL) | | Precision (RSD, %, n=6) | Repeatability (RSD, %, n=6) | Stability (RSD, %, n=10) | Accuracy (n=6) | |
| --- | --- | --- | --- | --- | --- | --- | --- | --- | --- | --- |
|  | Range (ng/mL) | Equation | R^2^ | LOD | LOQ |  |  |  | (%) | (RSD, %) |
| 1 | 19.76-1.011×10^4^ | y=0.1345x-0.8967 | 0.9968 | 6.755 | 10.49 | 1.4 | 6.7 | 8.3 | 101.0 | 8.4 |
| 2 | 1.967-1007 | y=0.03023x-0.03812 | 0.9947 | 1.379 | 1.967 | 9.0 | 14 | 10 | 103.7 | 6.5 |
| 3 | 1.953-1000 | y=0.06775x-0.05439 | 0.9957 | 0.9740 | 1.803 | 4.1 | 3.1 | 7.6 | 110.5 | 2.5 |
| 4 | 2.009-1028 | y=0.05125x-0.03411 | 0.9954 | 0.6660 | 2.009 | 5.1 | 6.8 | 6.4 | 105.1 | 4.4 |
| 5 | 39.06-2.000×10^4^ | y=0.07742x-0.5903 | 0.9957 | 7.795 | 16.79 | 1.7 | 6.3 | 3.8 | 88.9 | 2.0 |
| 6 | 1.985-1016 | y=0.02231x-0.01788 | 0.9980 | 0.801 | 1.985 | 7.8 | 11 | 14 | 101.1 | 7.4 |
| 8 | 1.930-988 | y=0.001337x-0.001140 | 0.9955 | 0.9840 | 1.452 | 8.2 | 12 | 7.4 | 100.7 | 4.9 |
| 9 | 1.911-978 | y=0.9456x-0.6378 | 0.9959 | 0.6750 | 1.911 | 2.3 | 8.5 | 5.5 | 109.1 | 2.1 |
| 10 | 10.25-5250 | y=0.06866x-0.1874 | 0.9962 | 2.815 | 5.022 | 1.8 | 8.2 | 3.3 | 110.6 | 2.8 |
| 11 | 1.938-992 | y=1.625x-0.6566 | 0.9961 | 0.4360 | 0.9020 | 0.7 | 8.7 | 7.2 | 106.7 | 1.7 |
| 12 | 1.985-1016 | y=1.415x-0.8319 | 0.9977 | 0.6220 | 1.160 | 1.3 | 9.1 | 8.8 | 105.0 | 1.3 |
| 13 | 11.23-5750 | y=0.01384x-0.04845 | 0.9976 | 3.789 | 6.552 | 2.2 | 5.9 | 14 | 103.9 | 5.2 |
| 14 | 48.81-2.499×10^4^ | y=0.03001x+0.2015 | 0.9910 | 0.6600 | 2.042 | 3.0 | 7.6 | 15 | 100.7 | 7.1 |

**Table S3** Results of matrix effect investigation

| No | Linearity 1(matrix) | | Linearity 2(blank) | | Ion inhibition rate(%) |
| --- | --- | --- | --- | --- | --- |
|  | Equation | R^2^ | Equation | R^2^ |  |
| 1 | y=0.08701x+17.85 | 0.9984 | y=0.08557x-0.3720 | 0.9969 | 1.7 |
| 2 | y=0.02409x+0.05154 | 0.9950 | y=0.02186x-0.01247 | 0.9902 | 10 |
| 3 | y=0.04747x+0.5841 | 0.9968 | y=0.04661x-0.02437 | 0.9933 | 1.8 |
| 4 | y=0.03199x+0.3556 | 0.9966 | y=0.03087x-0.01575 | 0.9920 | 3.6 |
| 5 | y=0.05842x+43.35 | 0.9959 | y=0.05707x-0.1152 | 0.9975 | 2.4 |
| 6 | y=0.01353x+0.008097 | 0.9945 | y=0.01260x-0.005184 | 0.9936 | 7.4 |
| 8 | y=0.0008194x+0.01609 | 0.9921 | y=0.0007125x-0.003196 | 0.9960 | 15 |
| 9 | y=0.4992x+5.305 | 0.9968 | y=0.4627x-0.1072 | 0.9946 | 7.9 |
| 10 | y=0.03080x+2.005 | 0.9963 | y=0.03009x-0.02380 | 0.9983 | 2.3 |
| 11 | y=0.5833x+3.367 | 0.9985 | y=0.5943x-0.1700 | 0.9978 | -1.8 |
| 12 | y=0.3600x+3.899 | 0.9989 | y=0.3704x-0.1114 | 0.9964 | -2.8 |
| 13 | y=0.002354x+0.4701 | 0.9943 | y=0.002743x+0.001478 | 0.9939 | -14 |
| 14 | y=0.002069x+5.361 | 0.9920 | y=0.002412x-0.06059 | 0.9975 | -14 |

**Table S4** Precision and stability of representative ions in serum QC sample

| Ion | Intra-day precision RSD/% | | |  | Inter-day precision RSD/% | | |  | Stability RSD/% | | |
| --- | --- | --- | --- | --- | --- | --- | --- | --- | --- | --- | --- |
|  | *t*_R_ (min) | *m/z* | Intensity |  | *t*_R_ (min) | *m/z* | Intensity |  | *t*_R_(min) | *m/z* | Intensity |
| 512.2750 (-) | 0 | 0 | 3.28 |  | 0.09 | 0 | 5.30 |  | 0.14 | 0 | 5.78 |
| 514.2936 (-) | 0.10 | 0 | 2.41 |  | 0.20 | 0 | 5.33 |  | 0.31 | 0 | 4.40 |
| 540.3384 (-) | 0.17 | 0 | 1.12 |  | 0.05 | 0 | 1.40 |  | 0.11 | 0 | 2.28 |
| 568.3688 (-) | 0.07 | 0 | 2.03 |  | 0.06 | 0 | 1.23 |  | 0.06 | 0 | 2.11 |
| 747.5739 (-) | 0.06 | 0 | 1.60 |  | 0.08 | 0 | 1.68 |  | 0.08 | 0 | 2.38 |
| 616.1727 (+) | 0.08 | 0 | 2.34 |  | 0.11 | 0 | 5.19 |  | 0.12 | 0 | 5.15 |
| 496.3483 (+) | 0.07 | 0 | 1.51 |  | 0.13 | 0 | 2.10 |  | 0.12 | 0 | 4.89 |
| 524.3760 (+) | 0.05 | 0 | 1.85 |  | 0.10 | 0 | 1.55 |  | 0.10 | 0 | 4.26 |
| 360.3347(+) | 0.04 | 0 | 1.37 |  | 0.12 | 0 | 1.22 |  | 0.08 | 0 | 5.20 |
| 725.5530 (+) | 0.07 | 0 | 5.82 |  | 0.08 | 0 | 2.94 |  | 0.08 | 0 | 5.09 |

**Table S5** Differential serum metabolites related to water-expelling effect of SZT and VSZT in MAE rats and their relative abundance in each group ($\bar{x}$±*s*, *n*=6)

| No. | Quasi-molecular ion | *m/z* | *t*_R_ (min) | Formula | Metabolite | VIP | *q* value | M vs NC | | M_VL vs M | | M_VH vs M | |
| --- | --- | --- | --- | --- | --- | --- | --- | --- | --- | --- | --- | --- | --- |
|  |  |  |  |  |  |  |  | T | FC | T | FC | T | FC |
| 1 | [M+H]^+^ | 468.3085 | 6.86 | C_22_H_46_NO_7_P | LysoPC(14:0/0:0) | 1.90 | 3.60E-08 | ↓^****^ | 0.32 | ↑^#^ | 1.88 | ↑ | 1.75 |
| 2 | [M+H]^+^ | 482.3242 | 7.39 | C_23_H_48_NO_7_P | LysoPC(15:0/0:0) | 2.28 | 0 | ↓^****^ | 0.28 | ↑^##^ | 1.43 | ↑^###^ | 1.63 |
| 3 | [M+H]^+^ | 496.3403 | 7.93 | C_24_H_50_NO_7_P | LysoPC(16:0/0:0) | 6.58 | 4.90E-07 | ↓^****^ | 0.68 | ↑^####^ | 1.31 | ↑^####^ | 1.26 |
| 4 | [M+FA-H]^-^ | 596.3925 | 9.88 | C_28_H_58_NO_7_P | LysoPC(20:0/0:0) | 2.18 | 1.05E-11 | ↓^****^ | 0.32 | ↑^#^ | 1.42 | ↑^##^ | 1.65 |
| 5 | [M+FA-H]^-^ | 624.4237 | 10.67 | C_30_H_62_NO_7_P | LysoPC(22:0/0:0) | 1.32 | 1.10E-11 | ↓^****^ | 0.37 | ↑^###^ | 1.36 | ↑^###^ | 1.34 |
| 6 | [M+FA-H]^-^ | 652.4545 | 11.35 | C_32_H_66_NO_7_P | LysoPC(24:0/0:0) | 1.53 | 5.36E-13 | ↓^****^ | 0.39 | ↑^####^ | 1.29 | ↑^###^ | 1.25 |
| 7 | [M+FA-H]^-^ | 538.3144 | 7.17 | C_24_H_48_NO_7_P | LysoPC(16:1(9Z)/0:0) | 3.29 | 1.08E-13 | ↓^****^ | 0.31 | ↑^#^ | 1.32 | ↑^##^ | 1.42 |
| 8 | [M+H]^+^ | 522.3559 | 8.21 | C_26_H_52_NO_7_P | LysoPC(18:1(11Z)/0:0) | 7.23 | 1.06E-10 | ↓^****^ | 0.54 | ↑ | 1.22 | ↑^##^ | 1.37 |
| 9 | [M+H]^+^ | 520.3388 | 7.56 | C_26_H_50_NO_7_P | LysoPC(18:2(9Z,12Z)/0:0) | 3.99 | 0.002984 | ↓^****^ | 0.64 | ↑ | 1.18 | ↑^##^ | 1.38 |
| 10 | [M+H]^+^ | 550.3863 | 9.15 | C_28_H_56_NO_7_P | LysoPC(20:1(11Z)) | 3.65 | 0 | ↓^****^ | 0.33 | ↑^##^ | 1.40 | ↑^###^ | 1.47 |
| 11 | [M+FA-H]^-^ | 586.3140 | 7.04 | C_28_H_48_NO_7_P | LysoPC(20:5(5Z,8Z,11Z,14Z,17Z)/0:0) | 1.05 | 5.15E-07 | ↓^****^ | 0.30 | ↑ | 1.10 | ↑^####^ | 1.81 |
| 2 | [M+FA-H]^-^ | 624.4237 | 10.67 | C_30_H_60_NO_7_P | LysoPC(22:1(13Z)/0:0) | 1.32 | 1.10E-11 | ↓^****^ | 0.24 | ↑^####^ | 1.49 | ↑^####^ | 1.64 |
| 13 | [M+FA-H]^-^ | 650.4386 | 10.76 | C_32_H_64_NO_7_P | LysoPC(24:1(15Z)/0:0) | 1.54 | 5.01E-16 | ↓^****^ | 0.22 | ↑^#^ | 1.34 | ↑^###^ | 1.51 |
| 14 | [M-H]^-^ | 452.2769 | 6.86 | C_21_H_44_NO_7_P | LysoPE(0:0/16:0) | 1.11 | 1.63E-07 | ↓^****^ | 0.31 | ↑^##^ | 1.71 | ↑^#^ | 1.54 |
| 15 | [M+FA-H]^-^ | 526.3140 | 7.40 | C_23_H_48_NO_7_P | LysoPE(0:0/18:0) | 2.63 | 6.84E-14 | ↓^****^ | 0.29 | ↑ | 1.39 | ↑^##^ | 1.69 |
| 16 | [M+FA-H]^-^ | 580.3604 | 8.70 | C_27_H_54_NO_7_P | LysoPE(0:0/22:1(13Z)) | 1.08 | 2.08E-13 | ↓^****^ | 0.39 | ↑ | 1.19 | ↑^#^ | 1.33 |
| 17 | [M-H]^-^ | 534.3549 | 9.15 | C_27_H_54_NO_7_P | LysoPE(22:1(13Z)/0:0) | 1.78 | 2.59E-15 | ↓^****^ | 0.38 | ↑^#^ | 1.17 | ↑^####^ | 1.25 |
| 18 | [M+Na]^+^ | 327.2283 | 9.00 | C_20_H_32_O_2_ | Arachidonic acid | 2.52 | 0.001277 | ↓^****^ | 0.64 | ↑^####^ | 1.51 | ↑^####^ | 1.54 |
| 19 | [M+Na]^+^ | 415.2815 | 7.09 | C_24_H_40_O_4_ | Deoxycholic acid | 1.31 | 9.41E-08 | ↓^**^ | 0.31 | ↑^##^ | 3.27 | ↑^#^ | 2.72 |
| 20 | [M-H]^-^ | 319.2269 | 6.96 | C_20_H_32_O_3_ | 19(S)-HETE | 4.31 | 8.02E-11 | ↓^****^ | 0.21 | ↑^####^ | 5.34 | ↑^####^ | 4.70 |
| 21 | [M-H]^-^ | 407.2792 | 5.38 | C_24_H_40_O_5_ | Cholic acid | 1.71 | 0.000112 | ↓^**^ | 0.38 | ↑^###^ | 3.07 | ↑^####^ | 3.46 |
| 22 | [M+Na-2H]^-^ | 634.2376 | 7.62 | C_25_H_45_FeN_6_O_8_ | Ferrioxamine B | 1.31 | 7.61E-09 | ↓^****^ | 0.35 | ↑^##^ | 1.55 | ↑^##^ | 1.49 |
| 23 | [2M+Na]^+^ | 563.3119 | 7.56 | C_18_H_22_O_2_ | Estrone | 1.78 | 3.44E-13 | ↓^****^ | 0.39 | ↑ | 1.11 | ↑^##^ | 1.54 |

**Table S5** continued

| No. | Quasi-molecular ion | *m/z* | *t*_R_ (min) | Formula | Metabolite | VIP | *q* value | M vs NC | | M_VL vs M | | M_VH vs M | |
| --- | --- | --- | --- | --- | --- | --- | --- | --- | --- | --- | --- | --- | --- |
|  |  |  |  |  |  |  |  | T | FC | T | FC | T | FC |
| 24 | [M+H-2H_2_O]^+^ | 118.0661 | 1.25 | C_8_H_11_NO_2_ | Dopamine | 1.13 | 4.04E-11 | ↓^****^ | 0.36 | ↑^#^ | 1.45 | ↑^##^ | 1.49 |
| 25 | [M+H]^+^ | 303.2326 | 6.96 | C_20_H_30_O_2_ | Eicosapentaenoic acid | 3.20 | 1.71E-14 | ↓^***^ | 0.25 | ↑^####^ | 6.67 | ↑^####^ | 6.28 |
| 26 | [M+Na]^+^ | 369.2039 | 4.63 | C_21_H_30_O_4_ | Cortexolone | 1.28 | 1.65E-07 | ↓^****^ | 0.41 | ↑^#^ | 1.40 | ↑^###^ | 1.62 |
| 27 | [M+H-2H_2_O]^+^ | 1117.7007 | 14.85 | C_57_H_104_N_2_O_21_ | Ganglioside GM3 (d18:1/16:0) | 1.46 | 1.63E-06 | ↓^****^ | 0.78 | ↑^##^ | 1.13 | ↑^####^ | 1.18 |
| 28 | [M+K]^+^ | 243.0207 | 1.25 | C_11_H_12_N_2_O_2_ | L-Tryptophan | 1.24 | 3.97E-10 | ↓^****^ | 0.64 | ↑^#^ | 1.20 | ↑^###^ | 1.35 |
| 29 | [M+H]^+^ | 271.2631 | 9.45 | C_17_H_34_O_2_ | Heptadecanoic acid | 1.76 | 1.06E-06 | ↑^****^ | 2.07 | ↓^##^ | 0.77 | ↓^####^ | 0.67 |
| 30 | [M+H-2H_2_O]^+^ | 335.1666 | 9.05 | C_19_H_30_O_5_S | Androsterone sulfate | 1.64 | 3.69E-05 | ↑^****^ | 1.60 | ↓^#^ | 0.84 | ↓^#^ | 0.81 |
| 31 | [M+2Na-H]^+^ | 723.6011 | 12.64 | C_47_H_82_O_2_ | CE(20:1(11Z)) | 2.36 | 3.83E-10 | ↑^****^ | 3.89 | ↓^####^ | 0.37 | ↓^####^ | 0.37 |
| 32 | [M+H-H_2_O]^+^ | 369.3514 | 12.65 | C_27_H_46_O | Cholesterol | 6.48 | 0.000152 | ↑^***^ | 3.43 | ↓^###^ | 0.33 | ↓^##^ | 0.36 |
| 33 | [M+2Na-H]^+^ | 325.2117 | 9.05 | C_18_H_32_O_2_ | Linoleic acid | 4.81 | 2.29E-09 | ↑^**^ | 3.86 | ↓^#^ | 0.66 | ↓^#^ | 0.60 |
| 34 | [2M+H]^+^ | 817.5752 | 13.01 | C_24_H_40_O_5_ | 1b,3a,12a-Trihydroxy-5b-  cholanoic acid | 3.08 | 5.89E-14 | ↑^****^ | 1.68 | ↓^####^ | 0.81 | ↓^####^ | 0.81 |
| 35 | [M+H-H_2_O]^+^ | 265.2513 | 9.02 | C_18_H_34_O_2_ | Oleic acid | 1.34 | 0 | ↑^****^ | 10.01 | ↓^#^ | 0.67 | ↓^##^ | 0.57 |
| 36 | [M+Na-2H]^-^ | 592.2648 | 7.60 | C_26_H_37_N_9_O_6_ | Kinetensin 4-7 | 1.48 | 1.97E-09 | ↑^****^ | 2.35 | ↓^####^ | 0.73 | ↓^###^ | 0.73 |
| 37 | [M+Cl]^-^ | 1110.5278 | 13.42 | C_50_H_73_N_15_O_12_ | Bradykinin hydroxyproline | 1.08 | 2.98E-10 | ↑^****^ | 4.68 | ↓^###^ | 0.63 | ↓^##^ | 0.73 |
| 38 | [M-H]^-^ | 722.4421 | 14.87 | C_37_H_70_NO_8_P | PE(14:0/18:2(9Z,12Z)) | 1.33 | 3.43E-14 | ↑^****^ | 7.41 | ↓^###^ | 0.63 | ↓^####^ | 0.52 |
| 39 | [M+FA-H]^-^ | 806.5919 | 13.57 | C_42_H_84_NO_8_P | PE(15:0/22:0) | 5.36 | 2.70E-12 | ↑^****^ | 2.41 | ↓^##^ | 0.79 | ↓^###^ | 0.72 |
| 40 | [2M-H]^-^ | 667.4239 | 14.59 | C_20_H_30_O_4_ | Prostaglandin J2 | 1.34 | 6.95E-14 | ↑^****^ | 6.65 | ↓^##^ | 0.71 | ↓^###^ | 0.55 |
| 41 | [M+K]^+^ | 319.1947 | 9.05 | C_18_H_32_O_2_ | 9E,11E-Octadecadienoic acid | 8.36 | 1.01E-08 | ↑^****^ | 2.08 | ↓ | 0.89 | ↓ | 0.88 |
| 42 | [M-H]^-^ | 514.2835 | 5.21 | C_26_H_45_NO_7_S | Taurocholic acid | 10.07 | 4.37E-11 | ↑^**^ | 18.27 | ↓ | 0.61 | ↓ | 0.75 |
| 43 | [2M-H]^-^ | 1123.7710 | 12.68 | C_33_H_54_O_7_ | Cholesterol glucuronide | 1.69 | 1.39E-06 | ↓^****^ | 0.57 | ↑ | 1.04 | ↑ | 1.15 |
| 44 | [M-H-H_2_O]^-^ | 974.2910 | 12.01 | C_35_H_62_N_7_O_18_P_3_S | (S)-3-Hydroxytetradecanoyl-CoA | 1.02 | 1.16E-06 | ↓^***^ | 0.52 | ↑ | 1.03 | ↓ | 0.92 |

Note: FC: fold change; T: Trend; Comapred with NC group: ***P*<0.01, ****P*<0.001, *****P*<0.0001; Compared with M group: ^#^*P*<0.05, ^##^*P*<0.01, ^###^*P*<0.001, ^####^*P*<0.0001.

**Table S6** Precision and stability of representative ions in urinary QC sample

| Ion | Intra-day precision RSD/% | | |  | Inter-day precision RSD/% | | |  | Stability RSD/% | | |
| --- | --- | --- | --- | --- | --- | --- | --- | --- | --- | --- | --- |
|  | *t*_R_ (min) | *m/z* | Intensity |  | *t*_R_ (min) | *m/z* | Intensity |  | *t*_R_ (min) | *m/z* | Intensity |
| 188.9891 (-) | 0 | 0 | 1.62 |  | 0 | 0 | 5.62 |  | 0 | 0 | 3.99 |
| 212.0106 (-) | 0.19 | 0 | 1.60 |  | 0.19 | 0 | 2.71 |  | 0.16 | 0 | 1.97 |
| 178.0560 (-) | 0.11 | 0 | 1.39 |  | 0.11 | 0 | 2.25 |  | 0.10 | 0 | 1.63 |
| 201.0275 (-) | 0 | 0 | 1.52 |  | 0.09 | 0 | 3.76 |  | 0.08 | 0 | 2.80 |
| 343.0897 (-) | 0.07 | 0 | 3.52 |  | 0.07 | 0 | 5.36 |  | 0.07 | 0 | 4.04 |
| 245.1639 (+) | 0.17 | 0 | 3.12 |  | 0.26 | 0 | 10.68 |  | 0.24 | 0 | 8.35 |
| 397.0688 (+) | 0.08 | 0 | 0.84 |  | 0.08 | 0 | 1.24 |  | 0.07 | 0 | 1.69 |
| 321.0975 (+) | 0.06 | 0 | 1.85 |  | 0.06 | 0 | 2.88 |  | 0 | 0 | 2.11 |
| 398.2441(+) | 0 | 0 | 2.46 |  | 0 | 0 | 4.65 |  | 0 | 0 | 3.28 |
| 360.3264 (+) | 0.05 | 0 | 1.64 |  | 0.03 | 0 | 1.55 |  | 0.05 | 0 | 1.73 |

**Table S7** Differential urinary metabolites related to water- expelling effect of SZT and VSZT in MAE rats and their relative abundance in each group ($\bar{x}$±*s*, *n*=6)

| No. | Quasi-molecular ion | *m/z* | *t*_R_ (min) | Formula | Metabolite | VIP | *q* value | M vs NC | | M_VL vs M | | M_VH vs M | |
| --- | --- | --- | --- | --- | --- | --- | --- | --- | --- | --- | --- | --- | --- |
|  |  |  |  |  |  |  |  | T | FC | T | FC | T | FC |
| 1 | [M-H]^-^ | 217.0818 | 1.46 | C_8_H_14_N_2_O_5_ | 5-L-Glutamyl-L-alanine | 1.13 | 0 | ↑^****^ | 9.15 | ↓^#^ | 0.74 | ↓^####^ | 0.48 |
| 2 | [2M+FA-H]^-^ | 357.0294 | 1.53 | C_5_H_4_N_2_O_4_ | Orotic acid | 3.11 | 7.14E-11 | ↑^****^ | 7.64 | ↓^####^ | 0.44 | ↓^####^ | 0.40 |
| 3 | [M-H]^-^ | 201.1126 | 7.66 | C_10_H_18_O_4_ | Heptylmalonic acid | 2.69 | 1.00E-05 | ↑^****^ | 1.85 | ↓^####^ | 0.63 | ↓^####^ | 0.56 |
| 4 | [2M-H]^-^ | 275.0574 | 4.98 | C_7_H_6_O_3_ | Gentisate aldehyde | 1.26 | 2.55E-05 | ↑^***^ | 1.86 | ↓^####^ | 0.29 | ↓^####^ | 0.40 |
| 5 | [M-H]^-^ | 350.1078 | 1.63 | C_13_H_21_NO_10_ | N-Acetyl-4-*O*-acetylneuraminic acid | 2.86 | 0 | ↑^****^ | 5.26 | ↓^#^ | 0.71 | ↓^####^ | 0.65 |
| 6 | [M-H]^-^ | 350.1078 | 1.23 | C_13_H_21_NO_10_ | N-Acetyl-7-*O*-acetylneuraminic acid | 3.25 | 0 | ↑^****^ | 4.14 | ↓^#^ | 0.71 | ↓^###^ | 0.68 |
| 7 | [M-H]^-^ | 199.0969 | 7.27 | C_10_H_16_O_4_ | Decenedioic acid | 5.28 | 4.46E-06 | ↑^****^ | 5.59 | ↓^####^ | 0.43 | ↓^####^ | 0.43 |
| 8 | [M+H]^+^ | 169.0369 | 1.53 | C_5_H_4_N_4_O_3_ | Uric acid | 13.21 | 2.87E-05 | ↑^****^ | 2.79 | ↓^####^ | 0.50 | ↓^####^ | 0.55 |
| 9 | [M-H]^-^ | 267.0728 | 2.75 | C_10_H_12_N_4_O_5_ | Inosine | 2.84 | 2.73E-05 | ↑^****^ | 4.35 | ↓^####^ | 0.44 | ↓^####^ | 0.34 |
| 10 | [M+FA-H]^-^ | 271.0560 | 1.59 | C_9_H_10_N_2_O_5_ | 3-Nitrotyrosine | 1.50 | 2.11E-11 | ↑^****^ | 3.69 | ↓^#^ | 0.76 | ↓^###^ | 0.62 |
| 11 | [M-H]^-^ | 155.1074 | 7.27 | C_9_H_16_O_2_ | 4-Hydroxynonenal | 2.49 | 3.60E-07 | ↑^****^ | 4.36 | ↓^####^ | 0.50 | ↓^####^ | 0.43 |
| 12 | [M+FA-H]^-^ | 203.1274 | 6.50 | C_9_H_18_O_2_ | Pelargonic acid | 1.16 | 5.31E-05 | ↑^****^ | 4.34 | ↓^####^ | 0.50 | ↓^####^ | 0.42 |
| 13 | [M+FA-H]^-^ | 164.0571 | 1.66 | C_4_H_9_NO_3_ | L-Threonine | 1.82 | 3.28E-11 | ↑^****^ | 3.16 | ↓ | 0.83 | ↓^###^ | 0.69 |
| 14 | [2M-H]^-^ | 225.1114 | 7.67 | C_4_H_7_N_3_O | Creatinine | 1.19 | 0.000266 | ↑^****^ | 2.10 | ↓^####^ | 0.52 | ↓^####^ | 0.38 |
| 15 | [M-H_2_O-H]^-^ | 153.0300 | 1.24 | C_6_H_8_N_2_O_4_ | Hydantoin-5-propionic acid | 4.29 | 5.84E-11 | ↑^****^ | 2.95 | ↓^##^ | 0.88 | ↓^####^ | 0.69 |
| 16 | [2M+FA-H]^-^ | 465.1478 | 7.28 | C_8_H_10_N_4_O_3_ | 1,3,7-Trimethyluric acid | 1.52 | 3.79E-07 | ↑^****^ | 3.74 | ↓^####^ | 0.44 | ↓^####^ | 0.41 |
| 17 | [M+FA-H]^-^ | 183.0398 | 1.24 | C_6_H_6_N_2_O_2_ | Urocanic acid | 2.02 | 5.21E-11 | ↑^****^ | 2.59 | ↓ | 0.88 | ↓^####^ | 0.72 |
| 18 | [M+FA-H]^-^ | 225.0871 | 3.48 | C_9_H_12_N_2_O_2_ | 5-Hydroxykynurenamine | 2.53 | 7.08E-14 | ↑^****^ | 2.38 | ↓^##^ | 0.79 | ↓^####^ | 0.72 |
| 19 | [M+Na-2H]^-^ | 210.0871 | 1.00 | C_7_H_15_N_3_O_3_ | Homocitrulline | 1.35 | 1.88E-07 | ↑^****^ | 3.14 | ↓^##^ | 0.76 | ↓^####^ | 0.61 |
| 20 | [M-H]^-^ | 229.1436 | 7.28 | C_12_H_22_O_4_ | Dodecanedioic acid | 1.98 | 5.07E-05 | ↑^****^ | 3.12 | ↓^####^ | 0.44 | ↓^####^ | 0.47 |
| 21 | [2M+FA-H]^-^ | 371.1322 | 5.35 | C_6_H_13_NO_2_S | S-(2-carboxypropyl)-Cysteamine | 2.36 | 7.66E-05 | ↑^**^ | 2.17 | ↓^###^ | 0.34 | ↓^###^ | 0.46 |
| 22 | [2M-H]^-^ | 363.0771 | 5.87 | C_6_H_6_N_4_O_3_ | 3-Methyluric acid | 1.43 | 2.21E-05 | ↑^***^ | 1.93 | ↓^###^ | 0.52 | ↓^###^ | 0.53 |
| 23 | [M+Cl]^-^ | 401.1730 | 5.56 | C_20_H_30_O_6_ | 20-Carboxyleukotriene B4 | 2.10 | 4.21E-08 | ↑^****^ | 2.89 | ↓^####^ | 0.42 | ↓^####^ | 0.58 |

**Table S7** continued

| No. | Quasi-molecular ion | *m/z* | *t*_R_ (min) | Formula | Metabolite | VIP | *q* value | M vs NC | | M_VL vs M | | M_VH vs M | |
| --- | --- | --- | --- | --- | --- | --- | --- | --- | --- | --- | --- | --- | --- |
|  |  |  |  |  |  |  |  | T | FC | T | FC | T | FC |
| 24 | [M+Na-2H]^-^ | 267.1216 | 7.54 | C_12_H_22_O_5_ | 3-Hydroxydodecanedioic acid | 1.50 | 2.70E-06 | ↑^****^ | 2.14 | ↓ | 0.95 | ↓^##^ | 0.68 |
| 25 | [M+FA-H]^-^ | 289.0675 | 7.28 | C_9_H_12_N_2_O_6_ | Pseudouridine | 1.84 | 1.25E-06 | ↑^****^ | 2.57 | ↓^##^ | 0.72 | ↓^####^ | 0.61 |
| 26 | [M-H]^-^ | 512.2653 | 8.21 | C_26_H_43_NO_7_S | Sulfolithocholylglycine | 1.04 | 2.13E-07 | ↑^****^ | 12.51 | ↓^####^ | 0.49 | ↓^####^ | 0.41 |
| 27 | [M+Na]^+^ | 305.0849 | 3.30 | C_11_H_14_N_4_O_5_ | 1-Methylinosine | 3.39 | 3.69E-06 | ↑^****^ | 1.73 | ↓^####^ | 0.52 | ↓^####^ | 0.48 |
| 28 | [M+H]^+^ | 206.0457 | 4.65 | C_10_H_7_NO_4_ | Xanthurenic acid | 8.41 | 0.000475 | ↑^****^ | 2.13 | ↓^####^ | 0.51 | ↓^####^ | 0.44 |
| 29 | [M+K]^+^ | 230.0129 | 5.45 | C_10_H_9_NO_3_ | 5-Hydroxyindoleacetic acid | 1.38 | 3.20E-05 | ↑^***^ | 1.51 | ↓^####^ | 0.43 | ↓^####^ | 0.57 |
| 30 | [M+H]^+^ | 112.0512 | 3.57 | C_4_H_5_N_3_O | Cytosine | 1.75 | 7.50E-06 | ↑^****^ | 7.24 | ↓^####^ | 0.42 | ↓^####^ | 0.35 |
| 31 | [M+H-H_2_O]^+^ | 130.0653 | 2.75 | C_9_H_9_NO | 3-Methylene-indolenine | 1.35 | 2.24E-09 | ↑^****^ | 2.97 | ↓^####^ | 0.53 | ↓^####^ | 0.46 |
| 32 | [2M+Na]^+^ | 255.0831 | 4.52 | C_5_H_8_O_3_ | L-Phosphoarginine | 3.31 | 4.72E-05 | ↑^****^ | 2.51 | ↓^####^ | 0.32 | ↓^####^ | 0.49 |
| 33 | [M+H]^+^ | 218.1377 | 2.59 | C_10_H_19_NO_4_ | Propionylcarnitine | 1.37 | 1.13E-06 | ↑^****^ | 4.28 | ↓^####^ | 0.46 | ↓^#^ | 0.67 |
| 34 | [2M+Na]^+^ | 589.1703 | 3.83 | C_10_H_13_N_5_O_5_ | 8-Hydroxydeoxyguanosine | 1.28 | 0.001698 | ↑^***^ | 1.86 | ↓^####^ | 0.40 | ↓ | 0.80 |
| 35 | [M+K]^+^ | 407.1844 | 4.11 | C_20_H_32_O_6_ | 6-Ketoprostaglandin E1 | 2.80 | 3.69E-10 | ↑^***^ | 2.79 | ↓ | 0.78 | ↓^##^ | 0.57 |
| 36 | [M+H]^+^ | 655.2751 | 9.56 | C_36_H_38_N_4_O_8_ | Coproporphyrin I | 6.12 | 8.39E-05 | ↑^****^ | 13.99 | ↓^####^ | 0.19 | ↓^##^ | 0.39 |
| 37 | [M+Na]^+^ | 588.5335 | 13.88 | C_36_H_71_NO_3_ | Ceramide (d18:1/18:0) | 1.08 | 3.70E-06 | ↑^**^ | 7.67 | ↓^##^ | 0.26 | ↓^##^ | 0.29 |
| 38 | [M+H]^+^ | 268.1038 | 2.63 | C_10_H_13_N_5_O_4_ | Adenosine | 4.11 | 1.42E-05 | ↑^****^ | 4.01 | ↓^####^ | 0.37 | ↓^####^ | 0.23 |
| 39 | [M+H]^+^ | 384.1160 | 3.94 | C_14_H_17_N_5_O_8_ | Succinyladenosine | 12.76 | 1.40E-14 | ↑^****^ | 4.75 | ↓ | 0.72 | ↓^##^ | 0.69 |
| 40 | [M+H_2_O-H]^-^ | 142.0657 | 6.12 | C_10_H_11_NO | Tryptophanol | 1.28 | 1.17E-05 | ↓^****^ | 0.23 | ↑^#^ | 1.89 | ↑^####^ | 4.48 |
| 41 | [M+H-H_2_O]^+^ | 170.0614 | 6.12 | C_11_H_9_NO_2_ | Indoleacrylic acid | 8.10 | 9.85E-06 | ↓^****^ | 0.17 | ↑^#^ | 2.44 | ↑^####^ | 4.68 |
| 42 | [M-H_2_O-H]^-^ | 175.0245 | 3.01 | C_6_H_10_O_7_ | D-Glucuronic acid | 4.32 | 2.10E-07 | ↓^****^ | 0.21 | ↓ | 0.70 | ↑ | 1.46 |
| 43 | [M-H]^-^ | 159.0659 | 5.86 | C_7_H_12_O_4_ | 3,3-Dimethylglutaric acid | 1.44 | 1.20E-09 | ↓^****^ | 0.31 | ↓ | 0.66 | ↓ | 0.99 |
| 44 | [2M+FA-H]^-^ | 215.1024 | 3.12 | C_4_H_7_NO | 2-Pyrrolidinone | 1.67 | 3.34E-08 | ↓^****^ | 0.33 | ↓ | 0.85 | ↑ | 1.11 |
| 45 | [M+H]^+^ | 286.1378 | 4.23 | C_12_H_19_N_3_O_5_ | Glycylprolylhydroxyproline | 1.11 | 6.11E-07 | ↓^****^ | 0.53 | ↓ | 0.64 | ↑ | 1.02 |
| 46 | [M+Na]^+^ | 184.0376 | 4.92 | C_9_H_7_NO_2_ | 4,6-Dihydroxyquinoline | 2.24 | 1.56E-06 | ↓^****^ | 0.30 | ↑ | 1.04 | ↑ | 1.20 |

Note: FC: fold change; T: Trend; Comapred with NC group: ***P*<0.01, ****P*<0.001, *****P*<0.0001; Compared with M group: ^#^*P*<0.05, ^##^*P*<0.01, ^###^*P*<0.001, ^####^*P*<0.0001

**Table S8** Bacterial network attribute parameters

| Parameters | NC | M | M_VL | M_VH |
| --- | --- | --- | --- | --- |
| Nodes | 334 | 348 | 376 | 354 |
| Edges | 1091 | 1258 | 2621 | 1383 |
| Positive edges | 811 | 1077 | 1868 | 940 |
| Negative edges | 280 | 181 | 753 | 443 |
| Average degree | 6.533 | 7.23 | 13.941 | 7.814 |
| Network diameter | 10 | 11 | 6 | 10 |
| Graph density | 0.02 | 0.021 | 0.037 | 0.022 |
| Connected components | 12 | 13 | 8 | 13 |
| Modularity | 0.802 | 0.778 | 0.686 | 0.724 |
| Average clustering coeffcient | 0.536 | 0.573 | 0.545 | 0.536 |
| Average path length | 4.559 | 4.284 | 3.167 | 4.196 |

**Table S9** The precursor ion, product ion and collision energy for each SCFA.

| Analytes | Precursor ion (*m*/*z*) | Product ion (*m*/*z*) | Collision energy (eV) |
| --- | --- | --- | --- |
| Acetic acid | 60 | 45 | 10 |
| Propionic acid | 74 | 73 | 5 |
| Butyric acid | 60 | 42 | 10 |
| Valeric acid | 60 | 42 | 10 |
| Isobutyric acid | 73 | 55 | 10 |
| Isovaleric acid | 60 | 42 | 10 |
| 2-methylvaleric acid | 74 | 73 | 5 |
