# Supplementary material for: Feasibility of vinegar processing of toxic herbs in Shi–Zao–Tang: toxicity reduction, efficacy preservation in malignant ascites rats and underlying pharmacodynamic mechanisms
Source: Chin Med. 2025 Oct 4;20:156. doi: 10.1186/s13020-025-01224-9 (PMC12495871; doi:10.1186/s13020-025-01224-9)
Supplement: Supplementary file 3 — Additional file 3. Fig. S1 Specaccum curve and Rank abundance curve of gut microbiota analysis (n = 6). (A) Specaccum curve. (B) Rank abundance curve. Fig. S2 Co-occurrence network analysis of microbial interactions at the genus level of NC, M, M_VL and M_VH group. The size of the node is directly proportional to the connectivity of the OUT. The red edge represents positive correlation, and the green edge represents negative correlation. Fig. S3 Plots of ZiPi analysis for bacteria in NC, M, M_VL and M_VH group (n = 6). [file 13020_2025_1224_MOESM3_ESM.pptx]

## Slide 1
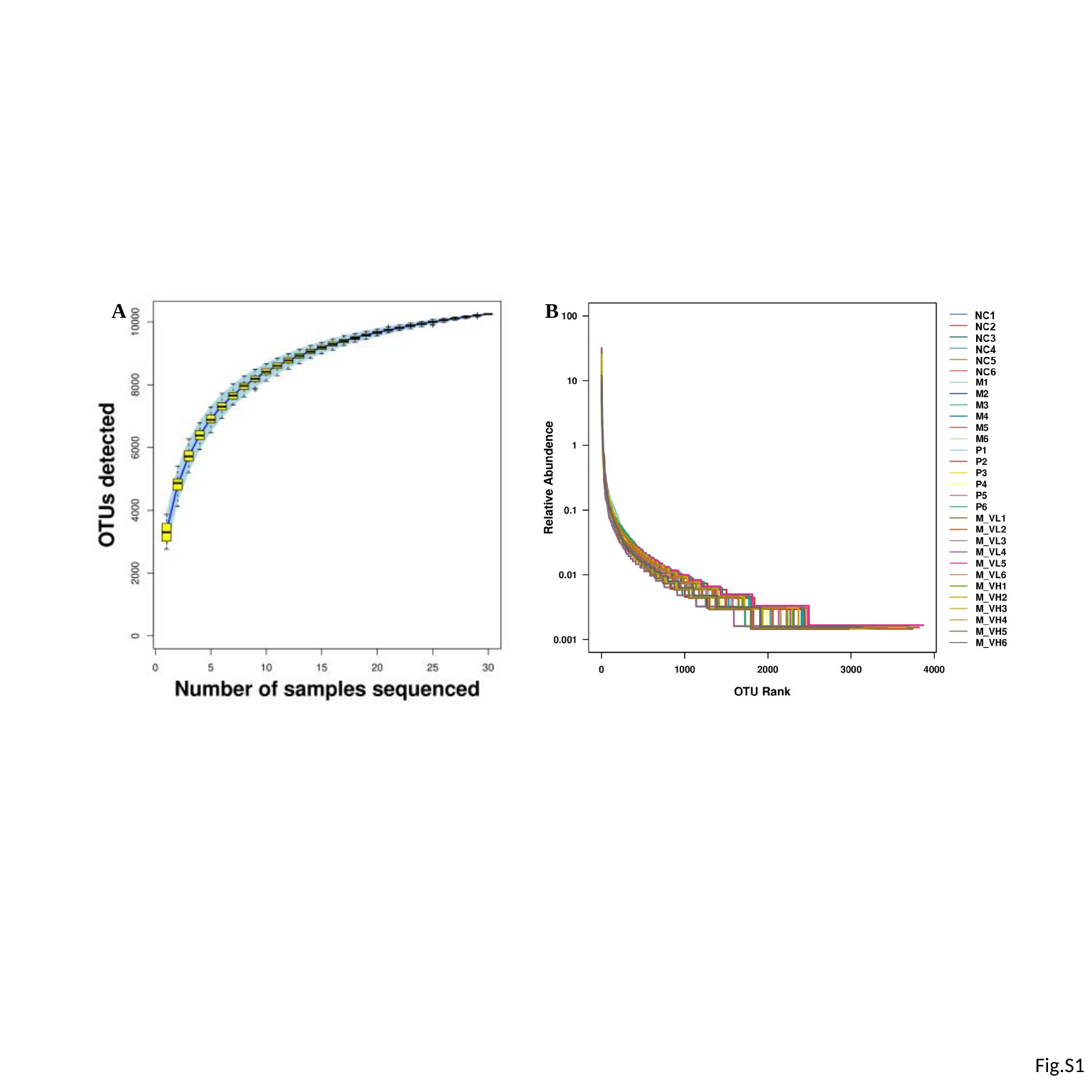

A
B
Fig.S1

## Slide 2
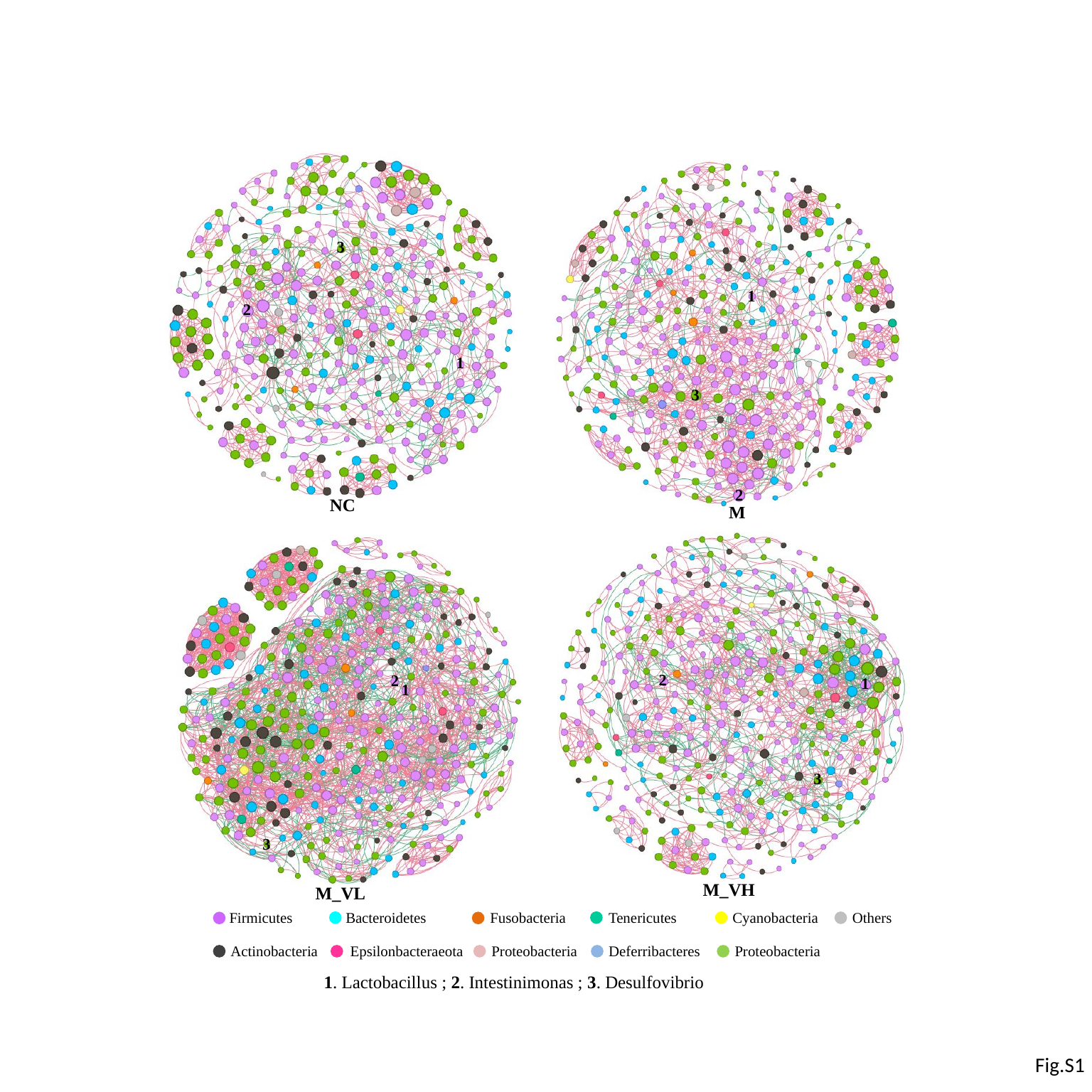

NC
M
M_VH
M_VL
Firmicutes
Bacteroidetes
Fusobacteria
Tenericutes
Cyanobacteria
Others
Actinobacteria
Epsilonbacteraeota
Proteobacteria
Deferribacteres
Proteobacteria
1. Lactobacillus ; 2. Intestinimonas ; 3. Desulfovibrio
Fig.S1

## Slide 3
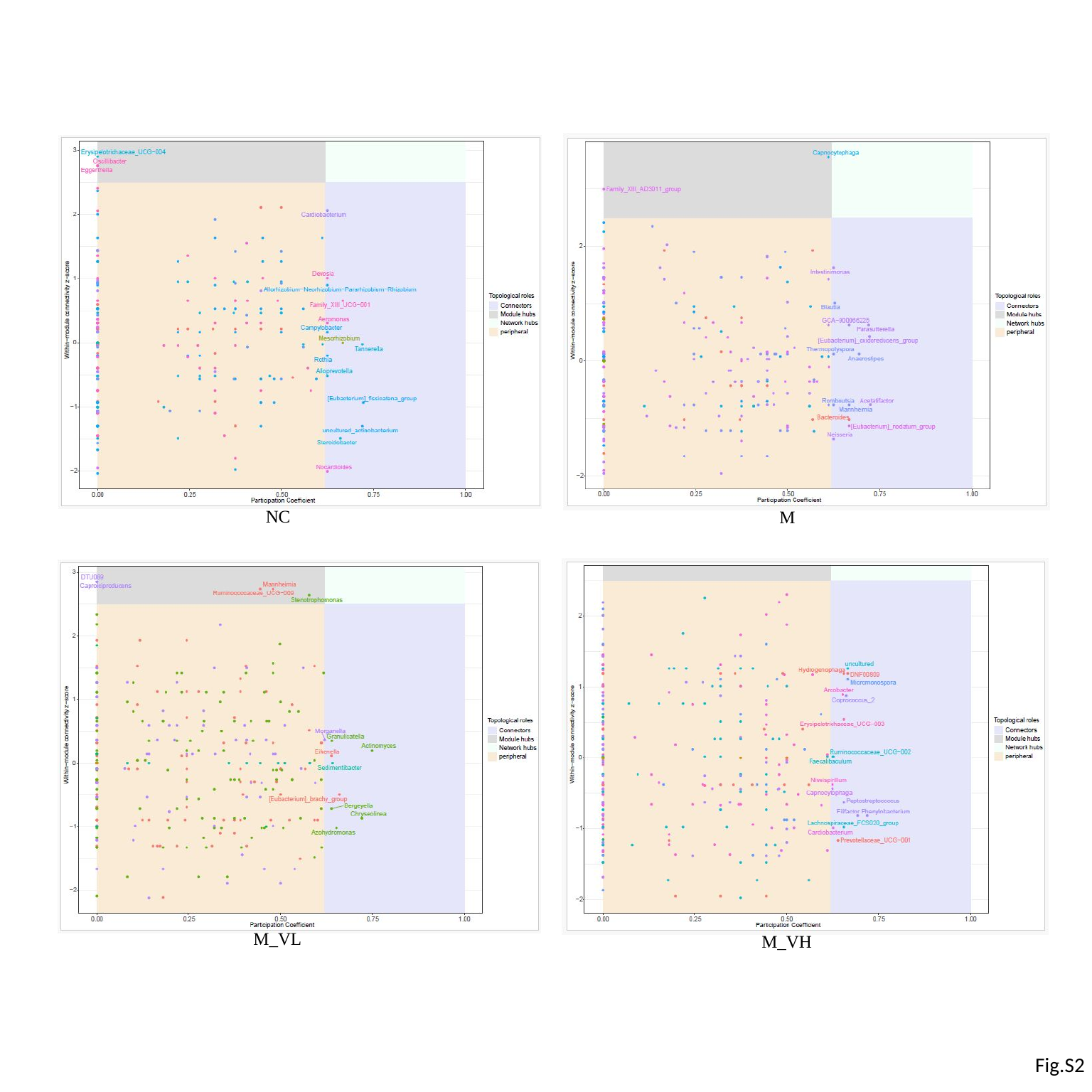

NC
M
M_VL
M_VH
Fig.S2
